# Targeted destruction of follicle stimulating hormone receptor-positive cancer cells in vitro and in vivo by a lytic peptide Phor21-FSHβ conjugate

**DOI:** 10.1186/s10020-025-01292-5

**Published:** 2025-06-09

**Authors:** Nafis A. Rahman, Marcin Chrusciel, Donata Ponikwicka-Tyszko, Kamila Pulawska-Moon, Milena Doroszko, Joanna Stelmaszewska, Oliver J. Keuzer, Adolfo Rivero-Muller, Piotr Bernaczyk, Grzegorz Zalewski, Peilan Guo, Jorma Toppari, Xiangdong Li, Adam J. Ziecik, Slawomir Wolczynski, Ilpo Huhtaniemi

**Affiliations:** 1 Institute of Biomedicine, University of Turku, Turku, Finland; 2 Department of Reproduction and Gynecological Endocrinology, Medical University of Bialystok, Bialystok, Poland; 3 Department of Biology and Pathology of Human Reproduction, Institute of Animal Reproduction and Food Research, Polish Academy of Sciences, Olsztyn, Poland; 4 Peptides & Elephants GmbH, Potsdam, Germany; 5 Department of Biochemistry and Molecular Biology, Medical University of Lublin, Lublin, Poland; 6 Department of Pathomorphology, Medical University of Bialystok, Bialystok, Poland; 7 State Key Laboratory of Agrobiotechnology, College of Biological Sciences, China Agricultural University, Beijing, China; 8 Centre for Population Health Research, and InFLAMES Research Flagship Center, University of Turku, Turku, Finland; 9 Department of Pediatrics, Turku University Hospital, Turku, Finland; 10 Department of Hormonal Action Mechanisms, Institute of Animal Reproduction and Food Research, Polish Academy of Sciences, Olsztyn, Poland; 11 Department of Metabolism, Digestion and Reproduction, Imperial College London, London, UK

**Keywords:** FSHR, Lytic peptide, Phor21, FSHβ, Cancer

## Abstract

**Background:**

Extragonadal follicle-stimulating hormone (FSH) receptor (FSHR) expression in various cancers and their endothelial vessel cells has highlighted novel opportunities for targeted FSHR therapy.

**Methods:**

We investigated the specificity/cytotoxicity of Phor21 fusion lytic peptide, conjugated to 12 different FSHβ-chain fragments to ablate FSHR-expressing cancer cells in vitro and *in vivo.* Additionally, the use of the gonadotropin-releasing hormone (GnRH) antagonist cetrorelix (CTX) alone or with the Phor21-FSHβ33-53 C/S conjugate for anticancer therapy was analyzed.

**Results:**

Phor21 linked to the FSHβ33–53 fragment with cysteine (Cys) replaced by serine (Ser) (Phor21-FSHβ33-53 C/S) demonstrated the highest specific cytotoxicity towards FSHR possessing cancer cells vs. other compounds. Recombinant human FSH treatment significantly decreased the cytotoxicity of Phor21-FSHβ33-53 C/S conjugate in FSHR-positive cancer cells. Phor21-FSHβ33-53 C/S (further addressed as Phor21-FSHβ) treatment in vivo significantly inhibited the growth of FSHR-positive cancer xenografts, resulting in necrosis. The efficacy of the Phor21-FSHβ was enhanced by co-treatment with the gonadotropin-releasing hormone (GnRH) antagonist cetrorelix (CTX). CTX alone exerted pro-apoptotic effects. CTX significantly inhibited the growth of prostate cancer LNCaP cell xenografts. Although FSHR-positive tumor vessel endothelial cells were previously reported in LNCaP cell xenografts, we were unable to reproduce FSHR expression. Consequently, Phor21-FSHβ had no effect on tumor destruction because of the lack of *Fshr* transcripts in the endothelium of these tumor vessel cells.

**Conclusion:**

This novel functional evidence shows that any cancer cell expressing FSHR can be specifically targeted and destroyed by the conjugated lytic peptide Phor21-FSHβ33–53 (Phor21-FSHβ). FSHR expression was not detected in the tumor vessel endothelial cells, which needs further re-evaluation.

**Graphical Abstract:**

Schematic overview of the Phor21-FSHb33-53C/S (Phor21-FSHβ) conjugate or CTX specifically targeted to kill FSHR-positive cancer cells. (Figure created using BioRender.com). Phor21-FSHb33-53C/S conjugate, Phor21 lytic backbone conjugated with a native or modified fragment of the FSHb subunit (FSHb33-53); CTX, GnRH antagonist cetrorelix

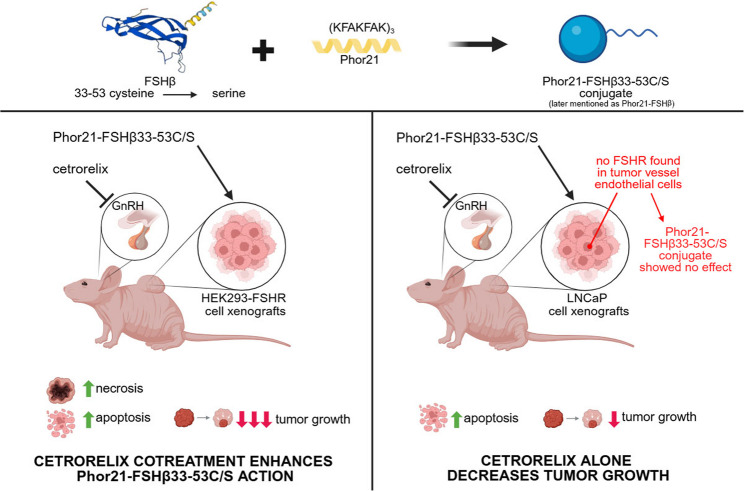

**Supplementary Information:**

The online version contains supplementary material available at 10.1186/s10020-025-01292-5.

## Highlights

Several cancers and their tumor vessel endothelial cells have been shown to express FSHR.

The fusion lytic peptide Phor 21 conjugated with the FSHβ chain 33–53 fragment (Phor21-FSHβ33-53 C/S) specifically targeted and destroyed cancer cells expressing FSHR.

We were unable to detect previously reported FSHR expression in tumor vessel endothelial cells, which may need further evaluation.

**Supplementary Information:**

The online version contains supplementary material available at 10.1186/s10020-025-01292-5.

## Introduction

Follicle-stimulating hormone (FSH) receptor (FSHR), a member of the G protein-coupled receptor family, is expressed in ovarian granulosa and testicular Sertoli cells (Griswold et al. 1995). Under pathological conditions, FSHR has been found in granulosa cell tumors (Chu et al. 2002), ovarian surface epithelial carcinomas (Wang et al. 2003; Bose 2008; Choi et al. 2004), ovarian Sertoli-Leydig cell tumors (Choong et al. 2002), prostate cancer (Ben-Josef et al. 1999; Mariani et al. 2006; Oduwole et al. 2021), endometrial cancer (Sheng et al. 2022), neuroendocrine tumors in the appendix (Starzynski et al. 2023), and normal fat tissue (Liu et al. 2015, 2017). FSHR is expressed in tumor vessel endothelial cells in the prostate, breast, colon, pancreas, urinary bladder, kidney, lung, liver, stomach, testis, and ovary (Planeix et al. 2015; Radu et al. 2010; Siraj et al. 2012), as well as in metastases (Siraj et al. 2013). In tumor vessel endothelial cells, FSHR may serve as a potential cellular tumor marker and novel target for cancer therapy (Ghinea 2010). The functional expression of FSHR has also been observed in deep endometriosis lesions (Ponikwicka-Tyszko et al. 2016). FSHR signaling was also found to promote angiogenesis in human umbilical vein endothelial cells (HUVECs) (Stilley et al. 2014a, b). However, this proangiogenic hypothesis is controversial, as contradictory results showing no functional FSHR expression in HUVECs have been published (Stelmaszewska et al. 2016). The transmembrane localization of FSHR and its highly specific affinity for its ligand make it a good target for the delivery of various therapeutic compounds.

Several membrane-disrupting analogs of naturally occurring lytic peptides, such as melittin [the main component of honeybee (*Apis mellifera*) venom], have been tested as anticancer agents (Rivero-Muller et al. 2007; Leuschner and Hansel 2004). Lytic peptides serve as defense molecules against microorganisms (e.g., bacteria and viruses), especially in insects with innate immunity (Leuschner and Hansel 2004). Although lytic peptides are diverse, they share many common characteristics, including linearity, total positive charge, alpha-helical structure, and amphipathicity in hydrophobic environments (Leuschner and Hansel 2004). These features enable them to bind to the negatively charged membranes of prokaryotic and eukaryotic cells (e.g., cancer cells). Examples of highly cytotoxic lytic peptides displaying increased activity against mammalian tumor cells include Phor14, Phor21, and Hecate (Bodek et al. 2003; Jia et al. 2008) Phor14 and Phor21 contain double or triple amino acid (AA) heptades KFAKFAK, respectively, whereas Hecate is a 23-AA synthetic analog of melittin. One of the major advantages of short lytic peptide conjugates is that they are not immunogenic and are rapidly metabolized (Jia et al. 2008; Bogacki et al. 2008). However, the rapid metabolism of conjugates does not affect their selectivity or efficiency in destroying cancer cells because they target them within minutes (Rivero-Muller et al. 2007; Bodek et al. 2005a, b).

The conjugation of lytic peptides with hormone ligands increases their specificity and selectivity for targeting cancer cells. In previous in vitro and in vivo studies, Phor14, Phor21, and Hecate lytic peptides were linked to a 15-amino acid fragment of the beta subunit of human chorionic gonadotropin (βCG) (Phor14-βCG, Phor21-βCG, and Hecate-βCG) to successfully target luteinizing hormone receptor (LHCGR)-expressing prostate (Jia et al. 2008; Bodek et al. 2005a, b; Hansel et al. 2001, 2007a, b), ovarian (Gawronska et al. 2002) and breast cancer cells (Bodek et al. 2003; Hansel et al. 2007a, b) or their metastases. Phor21-βCG(ala) has greater potency and ease of synthesis (Jia et al. 2008; Hansel et al. 2007a, b), therefore, has been chosen for further toxicity studies and tests in human trials and accepted into the National Cancer Institute Rapid Access to Intervention Development program for pharmacokinetic and cytotoxicity studies (Jia et al. 2008).

We hypothesized that FSHR-positive tumor cells or endothelial cells of tumor vessels could be targeted and destroyed by the Phor21 lytic peptide fused to a selected fragment of the FSHβ ligand. We analyzed the cytotoxicity of 12 different variants of the Phor21-FSHβ conjugate in vitro to select the peptide with the highest specific cytotoxicity against FSHR-expressing cells for in vivo xenograft studies. To exclude any possible competitive effects of endogenous FSH on Phor21-FSHβ treatment, we co-treated xenografted mice with a gonadotropin-releasing hormone (GnRH)-antagonist.

## Materials and methods

### Test compounds

The lytic peptide Phor21 backbone and Phor21-FSHβ conjugates were synthesized by Peptides and Elephants GmbH (Potsdam, Germany), using a TETRAS peptide synthesizer. Lytic peptide Phor 21 backbone and Phor 21-FSHβ conjugates were synthesized as one chain and additional conjugation process was not involved. The synthesized peptides were lyophilized and stored at −80 °C. The purities of the compounds were determined by HPLC analysis (Suppl Table 1).

### Cell culture

Human embryonic kidney 293 (HEK-293, RRID: CVCL_0045), human ovarian carcinoma (CaOV-3, RRID: CVCL_0201; OVCAR-3, RRID: CVCL_0465), human prostate carcinoma (PC-3, RRID: CVCL_0035; DU145, RRID: CVCL_0105 and LNCaP, RRID: CVCL_0395), and breast cancer MCF-7 (RRID: CVCL_0031) cell lines were obtained from the American Type Culture Collection (ATCC; Manassas, VA, USA). KGN human granulosa cancer cells (RRID: CVCL_0375) were kindly donated by Dr. T. Yanase (Graduate School of Medical Sciences, Kyushu University, Fukuoka, Japan). The HEK293-FSHR cell line was generated by stably transfecting HEK293 cells with FLAG-hFSHR/pcDNA3.1, c (2 kb) under the CMV promoter (Casarini et al. 2020; Jonas et al. 2018) kindly donated by Dr. A. C. Hanyaloglu (Imperial College, London, UK) using Lipofectamine^®^ LTX with Plus™ Reagent transfection reagent in Opti-MEM^®^ I reduced serum media (both Thermo Fisher Scientific Inc., UK). Cells harboring the FLAG-hFSHR/pcDNA3.1, plasmid were selected with 200 µg/ml Geneticin (G418) sulfate (Roche, Basel, Switzerland) in complete DMEM/F12 media. Following geneticin selection, the selected clones were tested for FSHR mRNA (by quantitative qPCR) and protein expression (by immunofluorescence staining). The function of transgenic FSHR in the HEK-293-FSHR cell line was tested via recombinant human (rh) FSH-stimulated cAMP production. All the cell lines were maintained in DMEM/F12 supplemented with 10% (v/v) fetal bovine serum (Thermo Fisher Scientific, Inc.), penicillin (100 IU/ml), and streptomycin (100 IU/ml) (Sigma-Aldrich, Saint Louis, Missouri, USA). The cells were incubated at 37 °C in a humidified atmosphere containing 5% CO2. Suspended cells were obtained using digestion solution containing 0.25% trypsin and 0.02% EDTA.

### Cytotoxicity test

Conjugate-mediated cytotoxicity in HEK293-FSHR and HEK-293 cells was determined using a CytoTox 96^®^ Assay (Promega, Madison, Wisconsin, USA), which quantitatively measures lactate dehydrogenase (LDH), a stable cytosolic enzyme released upon cell lysis. Before the experiment, the cells were seeded in a 96-well plate (15000 cells per/well in 100 µl of complete medium). After overnight incubation, the complete medium was replaced with stimulation medium, and the cells were treated for 1.5 h with the Phor21 backbone or Phor21-FSHβ conjugates at concentrations ranging from 0 to 5 µM. For competitive binding studies, the cells were pretreated with 100 IU/L rhFSH (7.3 ng/ml).

To visualize the cytotoxic effects of Phor21-FSHβ33–53 C/S in HEK293-FSHR cells, an in-house live/dead cell test was used. After Phor21-FSHβ33–53 C/S treatment for 15 min, the cells were simultaneously stained with green-fluorescent calcein-AM to indicate intracellular esterase activity (live cells) and with red-fluorescent propidium iodide to indicate loss of plasma membrane integrity (dead cells). Sample images were taken using a converted light microscope (EVOS, Thermo Fisher Scientific).

### Proliferation assay

Cell proliferation was determined using a CyQUANT Cell Proliferation Assay (C7026, Life Technologies, Thermo Fisher Scientific). Prior to the experiments, HEK-293, HEK293-FSHR, and LNCaP cells were seeded in 96-well plates (7000 cells/well). After overnight incubation in the culture medium, the cells were starved for 4 h in basal non-supplemented DMEM/F12. The cells were then treated for 48 h with rhFSH (10, 100, or 1000 IU/L) or cetrorelix (CTX) (1, 5, 10, or 20 µM; Sigma-Aldrich), and 2.5% FSC was used as a positive control. After incubation, the medium was removed, and the plates with cells were frozen at −80 °C overnight. After thawing at room temperature (RT), 200 µl of CyQUANT GR dye/cell lysis buffer was added to each well and incubated for 5 min at room temperature (RT) in the dark. The fluorescence was read using a WALLAC Victor 2 1420 spectrophotometer (Perkin Elmer, Waltham, Massachusetts, USA) with ~ 480 nm excitation and ~ 520 nm emission maxima. The results were subtracted from a standard curve made from bacteriophage λ DNA (ranging from 50 pg/ml to 1.0 µg/ml) in CyQUANT GR/cell lysis buffer and are presented as absolute values.

### Apoptosis assay

Caspase 3/7 activity was assessed using a Caspase Glo 3/7 kit (Promega) according to the manufacturer’s protocol. In brief, cells were seeded at 10,000/well in a 96-well plate, attached overnight, and treated with vehicle (CTR) or CTX (20 µM) for 12 h. Staurosporin (STS, 0.5 µM) was used as a positive inducer of caspase3/7-mediated apoptosis. The absolute luminescence was normalized to the vehicle-treated control value.

### Tumor xenografts

All the mouse experiments were approved by the local ethics committee of the Medical University of Bialystok, Poland. Athymic nude males (Crl: NU(NCr)-Foxn1^nu^, 8–10 weeks old, 20–23 g) purchased from Charles River Laboratories International, Inc. (Sulzfeld, Germany; RRID: SCR_003792) were housed two or three per cage and provided with sterilized pellet chow and water. The animals were maintained in a pathogen-free mouse colony at the Center of Experimental Medicine Animal Facility (Bialystok, Poland).

HEK293-FSHR, HEK-293, and LNCaP cells were treated with trypsin when they were nearly confluent and were harvested. The cells were centrifuged at 1100 rpm for 5 min and resuspended in sterile non-supplemented culture medium mixed 1:1 with the Matrigel^®^ matrix (Corning, Corning, USA). The cell suspension (1 × 10^6^ cells/0.2 ml - HEK293-FSHR and HEK-293 cells; 5 × 10^6^ cells/0.2 ml – LNCaP cells) was injected subcutaneously into the interscapulum area. When the xenograft volume reached 150–200 mm^3^ (5–8 days after implantation), the xenograft-bearing mice were randomly divided into treatment groups, as described in Suppl Table 2.

Three independent xenograft experiments were conducted. In the first set, we tested whether the Phor21-FSHβ33–53 C/S conjugate selectively targeted and destroyed FSHR-expressing HEK293-FSHR cells, using a proof-of-concept experiment. CTX was used to inhibit FSH secretion, which competed with Phor21-FSHβ33–53 C/S for FSHR-binding sites (Suppl Table 2). In the second set of experiments, we tested the specificity of the Phor21-FSHβ33–53 C/S conjugate using FSHR-negative HEK293 cells xenografted into nude mice (Suppl Table 2). Finally, in the third set of in vivo experiments, we tested whether Phor21-FSHβ33–53 C/S conjugate treatment could inhibit the growth of human prostate cancer LNCaP cell xenografts (FSHR-negative cells) by destroying FSHR-positive endothelial cells in xenograft tissues, as reported previously (Radu et al. 2010) (Suppl Table 2). Phor21-FSHβ33–53 C/S was later on addressed as Phor21-FSHβ.

In each experimental set, the mice were sacrificed 3 days after the last injection. Total blood was collected via heart puncture under isoflurane anesthesia. The animals were euthanized by cervical dislocation. Body weight, selected organs, xenograft weights/volumes, and sizes were recorded at the time of necropsy. The xenografts and testes were fixed in 10% neutral buffered formalin or snap-frozen in liquid nitrogen for further experiments.

### Total RNA isolation, reverse transcription and quantitative PCR

Total RNA from cell lines, xenografts, and mouse tissues was isolated using a standard protocol with the TRIzol reagent (Life Technologies, Thermo Fisher Scientific). Total RNA concentration was quantified using a NanoDrop 1000 spectrophotometer (NanoDrop Technologies, Wilmington, Delaware, USA). One microgram of RNA was treated with DNase I (Sigma‒Aldrich) and reverse-transcribed using the SensiFAST cDNA Synthesis Kit (Bioline, London, UK) at 48 °C for 60 min (for highly structured RNA).

Real-time quantitative polymerase chain reaction (qPCR) was performed using the DyNAmo HS SYBR Green qPCR Kit (Thermo Fisher Scientific) with the following primers:

PPIA (144 bp), F: GCCAAGACTGAGTGGTTGGATG; R: GAGTTGTCCACAGTCAGCAATGG; FSHR exon 1–2 (145 bp); F: TGGGCTCAGGATGTCATCATCGGA; R: TGGATGACTCGAAGCTTGGTGAGG; FSHR exon 5–7 (136 bp); F: AAGCACCTTCCAGATGTTCAC; R: TCAGCCATAGAATCACACTTTCA; GNRHR (gonadotropin releasing hormone receptor) (116 bp), F: CAGAGCCCTTTGCCCATAATA; R: TGGTTACTGACTCCTCCAAATG; GNRHR (103 bp), F: TGGCACAACTAACACCTACTG; R: GAGGTAGACACAAGCCTGAATTA. qPCR was performed using the CFX96 Touch™ Real-Time PCR Detection System (Bio-Rad, Hercules, California, USA).

### cAMP production

The kinetics of rhFSH-stimulated intracellular cAMP production in HEK293-FSHR and HEK-293 cells were determined via Cyclic AMP iNdirect detection via light emission from sensor cells (CANDLES) assay, as described previously (Trehan et al. 2014). The cells were co-cultured with GS-293 sensor cells in 12-well plates in complete DMEM/F12 medium for 24 h. Prior to the experiment, complete DMEM was replaced with freshly prepared assay medium containing a 1:1 ratio of DMEM/F12 and CO2-independent medium supplemented with 0.1% BSA (Gibco, Thermo Fisher Scientific, Waltham, Massachusetts, USA), 2% GloSensor cAMP reagent (Promega), and 100 µM 3-isobutyl-1-methylxanthine (IBMX; Sigma). The cells were equilibrated for 45 min at RT in the dark at a constant temperature of 25 °C in a plate reader (Victor, Perkin-Elmer-Wallac), and luminescence was measured for 5 min to obtain a basal measurement. Luminescence from individual wells was measured for 5 s for up to 1 h.

Endpoint extracellular cAMP production in PC-3, DU145, LNCaP, KGN, CaOV-3, OVCaR-3, MCF-7, HEK293-FSHR, and HEK-293 cells was determined according to the standard radioimmunoassay method using iodinated succinyl-cAMP (Harper and Brooker 1975). The cells were seeded onto 24-well plates (80000 cells/well) and grown overnight in culture medium. Before stimulation, the cells were starved for 12 h in serum-free medium. cAMP production increased with the addition of 3-isobutyl-1-methylxanthine (IBMX; Sigma), a competitive nonselective phosphodiesterase inhibitor, to the stimulation medium. The cells were stimulated for 1 h at 37 °C in a humidified atmosphere containing 5% CO_2_. In both assays, the cells were stimulated with or without 10, 100, or 1000 IU/L rhFSH or 10 µM forskolin (FRK), which was used as the positive control.

### RNAScope

In situ hybridization of formalin-fixed paraffin-embedded (FFPE) blocks of xenografts was performed using an RNAscope FFPE 2.0 HD Detection Kit Brown (Wang et al. 2012) (Advanced Cell Diagnostics (ACD), Hayward, California, USA, CAT# 310033), as previously described (Stelmaszewska et al. 2016). Briefly, 5 μm FFPE sections of xenograft tissues were pretreated under ‘standard’ conditions and incubated with the following prewarmed probes: a mouse *Fshr* probe (Cat No. 400461), a mouse von Willebrand factor probe (*Vwf)* (Cat No. 499111), positive control probes for low-abundance transcripts Mm-*Polr-2a*, #312471), and a negative control probe (*DapB*, ACD-310043) for 2 h at 40 °C in a HybEZ(TM) oven (ACD). The slides were washed twice with 1X wash buffer for 2 min. Subsequent hybridization amplifiers were applied for 30 min (AMP 1, 3, 5) or 15 min (AMP 2, 4, 6) and incubated at 40 °C in a HybEZ(TM) oven (AMPs) with 2 min washes between washes. To visualize the signal, an equal volume mixture of brown A and brown B was added to the sections and incubated at RT for 10 min. After double washing with ddH_2_O and counterstaining for 2 min, fresh 50% Gill’s hematoxylin slides (Vector Laboratories, Burlingame, CA, USA) were washed with ddH_2_O and dipped in 0.02% ammonia water for 20 s. Dehydration was performed using fresh ethanol (70% for 2 min, twice with 100% for 2 min) and xylene for 5 min. Slides were mounted using Pertex (Histolab Products AB, Gothenburg, Sweden).

### Immunocytochemistry

HEK293-FSHR cells were grown on Millicell EZ Slide 8-well glass (Merck Millipore, Darmstadt, Germany) overnight in culture medium, fixed with 4% paraformaldehyde in PBS (15 min, RT), and washed with PBS (3 × 5 min). The cells were incubated for 30 min with blocking solution (3% BSA in PBS with 0.05% Tween 20; PBST) at RT. Thereafter, the cells were incubated in a humidified chamber for 1 h at RT with anti-hFSHR (FSHR323; 5 µg/ml; kindly donated by Dr. Ghinea, INSERM, Paris, France; RRID: AB_2106698) and anti-FLAG (F7425, 1:300, Sigma‒Aldrich; RRID: AB_439687) antibodies diluted in the blocking solution. The washed cells were incubated with goat anti-rabbit, goat anti-mouse, or donkey anti-goat IgG conjugated with Alexa Fluor 488 or Alexa Fluor 594 (all diluted 1:250; Thermo Fisher Scientific) for 45 min in the dark (RT). The cell nuclei were visualized by DAPI staining (1 µg/ml in PBS). The slides were mounted in a mounting medium (101098–042; Vector Laboratories, Burlingame, California, USA).

### Immunohistochemistry

Formalin-fixed paraffin sections (5 μm) of HEK293-FSHR xenografts were deparaffinized, hydrated, and boiled in 10 mM citric acid (pH 6.0) for 15 min for antigen retrieval. Endogenous peroxidase activity was reduced by incubation with 3% H_2_O_2_ for 15 min at RT, and the sections were blocked with bovine serum albumin (3% BSA) for 1 h at RT. The sections were then incubated with primary anti-Ki67 (Dako; RRID: AB_2250503) and anti-cleaved PARP1 (Cell Signaling Technology; RRID: AB_331426) antibodies in a blocking solution overnight at 4 °C. The slides were washed 3x for 5 min in PBST, and a DAKO EnVision + HRP-conjugated system (Dako, Glostrup, Denmark) was used as the secondary antibody. The signal was visualized using 3’3-diaminobenzidine tetrahydrochloride (DAB; Dako). The sections were counterstained with Meyer’s hematoxylin for 30 s, washed, dehydrated, and mounted using Pertex (Histolab Products AB; Gothenburg, Sweden).

### FSH measurements

The plasma levels of FSH were analyzed via an immunofluorometric assay using the Delfia Enhancer system (PerkinElmer, Turku, Finland) according to the protocol described by van Casteren et al. (Casteren et al. 2000). The FSH assay sensitivity was 0.1 µg/L, with an intra-assay CV of 4.3% and an interassay CV of 9.8% at 4.8 µg/L. The rat FSH standard (a gift from Dr. Albert Parlow from the National Institute of Diabetes and Digestive and Kidney Diseases, Bethesda, Maryland, USA) was diluted in Diluent II buffer (DELFIA Diluent II, PerkinElmer) to concentrations ranging from 50 ng/ml to 0.02 ng/ml for rat FSH. Enhanced fluorescence signals (DELFIA Enhancement Solution; PerkinElmer) were measured using a Victor^2^ fluorometer (PerkinElmer).

### Serum biochemical assay

To quantify the serum levels of glucose, γ-glutamyltransferase (GGT), alanine aminotransferase (ALT) and aspartate aminotransferase (AST), biochemical assays were performed using Fuji Dri-Chem NX500 (Fujifilm Corporation^®^) according to the manufacturer’s instructions (Lei et al. 2021).

### Statistical analysis

All numerical data are presented as the mean ± SEM. One-way ANOVA with Dunnett’s multiple comparison test (95% confidence interval) was used to analyze the statistical significance. Statistical analysis and graphs were prepared using GraphPad PRISM 6 (GraphPad Software Inc., San Diego, CA, USA; RRID: SCR_002798), and differences were considered significant at *P* ≤ 0.05.

## Results

### Phor21-FSHβ conjugates

We tested 12 variants of lytic Phor21-FSHβ conjugates (Suppl Table 1). The designed peptides were composed of a highly potent and easy to synthesize Phor21 lytic backbone (Jia et al. 2008) (KFAKFAKKFAKFAKKFAKFAK) conjugated with a native or modified fragment of the FSHβ subunit (FSHβ33–53 YTRDLVYKDPARPKIQKTCTF and FSHβ81-95QCHCGKCDSDSTDCT) or their combination (FSHβ33–53 + 81-95YTRDLVYKDPARPKIQKTCTFQCHCGKCDSDSTDCT). To increase the stability of the conjugates without losing their affinity for FSHR, cysteine residues in the FSHβ33–55 and 85–91 fragments were replaced with serine (C/S) or alanine (C/A) or stabilized by an acetamidomethyl group (Acm) (Suppl Table 1). The C-terminal was potentially used for coupling to enhance the stability and ease of synthesis, which does not affect the binding strength (capacity) of the peptide for the receptor.

### HEK293-FSHR cell line – an in vitro model for conjugate-mediated cytotoxicity analysis

**Fig. 1:**
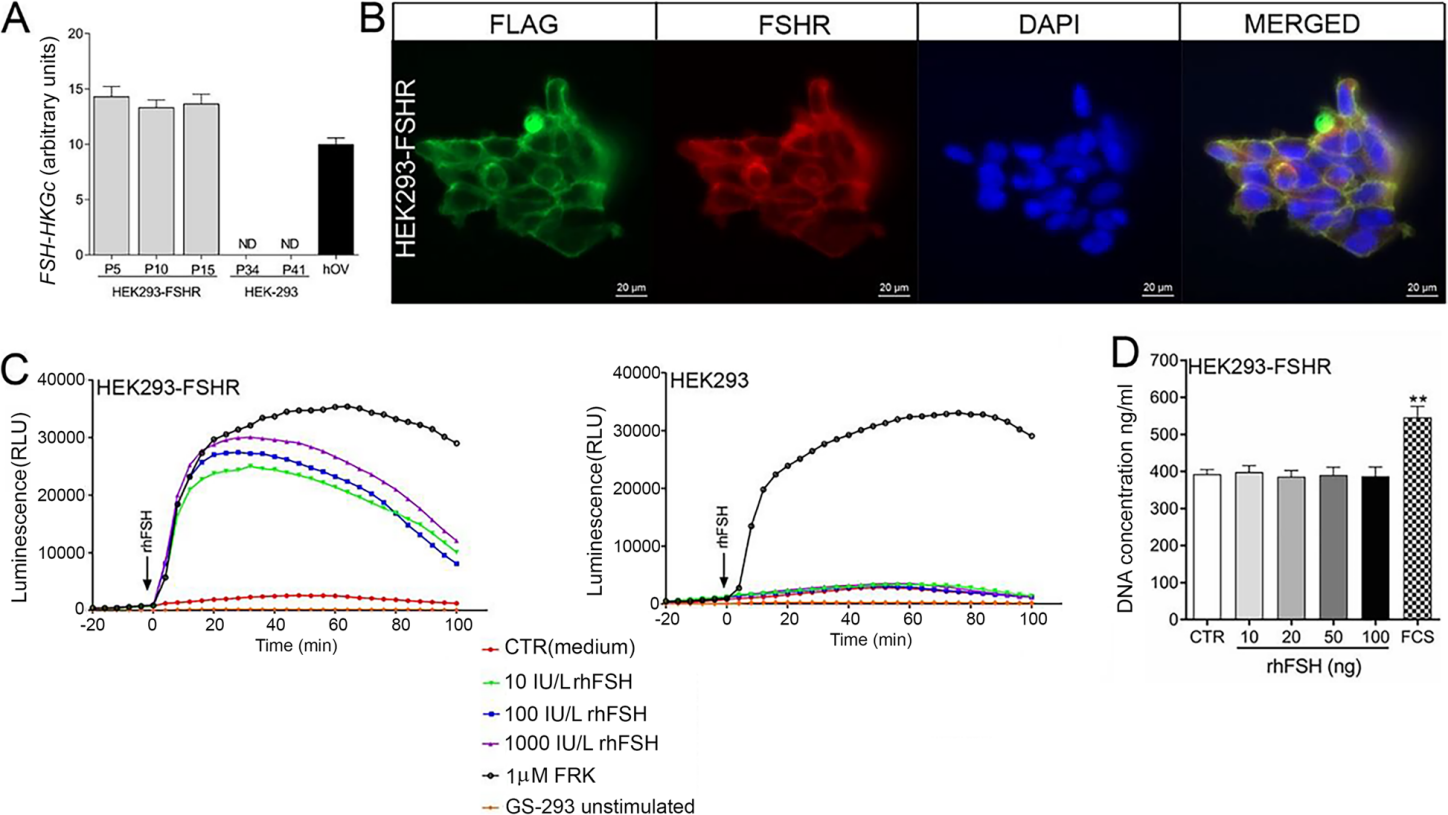
Functional characterization of the HEK293-FSHR cells. **A** qPCR relative expression of FSHR over 15 passages in HEK293-FSHR cells. HEK293 cells were used as a negative control, whereas the human ovary (OV) was used as a positive control for FSHR expression. Each bar represents the mean ±SEM of relative gene expression run in triplicate. **B** Immunofluorescence colocalization of human FSHR and FLAG in HEK293-FSHR cells. **C** Kinetics of rhFSH-stimulated cAMP production in HEK293-FSHR and control HEK293 cells, as determined by the CANDLES assay. **D** Effect of rhFSH on HEK293-FSHR cell proliferation. Fetal calf serum (FCS 2.5%) was used as a positive control stimulant for proliferation. Each bar represents the mean ±SEM of three independent experiments (*n* = 8/experiment). Asterisks indicate significant differences (**P ≤ 0.01) between control and stimulated cells. CANDLES, Cyclic AMP iNdirect detection by light emission from sensor cell assay; FLAG, polypeptide protein tag; FSHR, follicle-stimulating hormone receptor; HEK293, human embryonic kidney 293 cell line; HEK293-FSHR, HEK293 cells stably transfected with FLAG-hFSHR/pcDNA3.1; OV, ovary; rhFSH, recombinant human follicle-stimulating hormone; FCS, fetal calf serum; FRK, forskolin

Among the analyzed cancer cell lines, only KGN and human FSHR cDNA-stably transfected HEK293 (HEK293-FSHR) cells expressed functional FSHR (Fig. S1 A, B). However, *qPCR* analysis of KGN cells revealed that *FSHR* expression was rather unstable, as it decreased significantly (approximately 60%) at higher passage numbers (Fig. S2). Therefore, Phor21-FSHβ conjugate-mediated cytotoxicity was tested in HEK293-FSHR cells that were stably transfected with FSHR. The FLAG peptide sequence was used as a reporter for transgene expression in HEK293-FSHR cells. Characterization of the HEK293-FSHR cell line revealed stable expression of *FSHR* between passages (Fig. 1A), membrane colocalization of FSHR and FLAG (Fig. 1B) and dose-dependent rhFSH-stimulated cAMP production (Fig. 1C). However, rFSH stimulation did not promote HEK293-FSHR cell growth (Fig. 1D). Wild-type HEK-293 cells were used as FSHR-negative controls (Fig. 1C and Fig. S1).

### Phor21-FSHβ 33–55 C/S (further mentioned as Phor21-FSHβ) displayed the highest specific cytotoxicity toward FSHR-positive cells

**Fig. 2:**
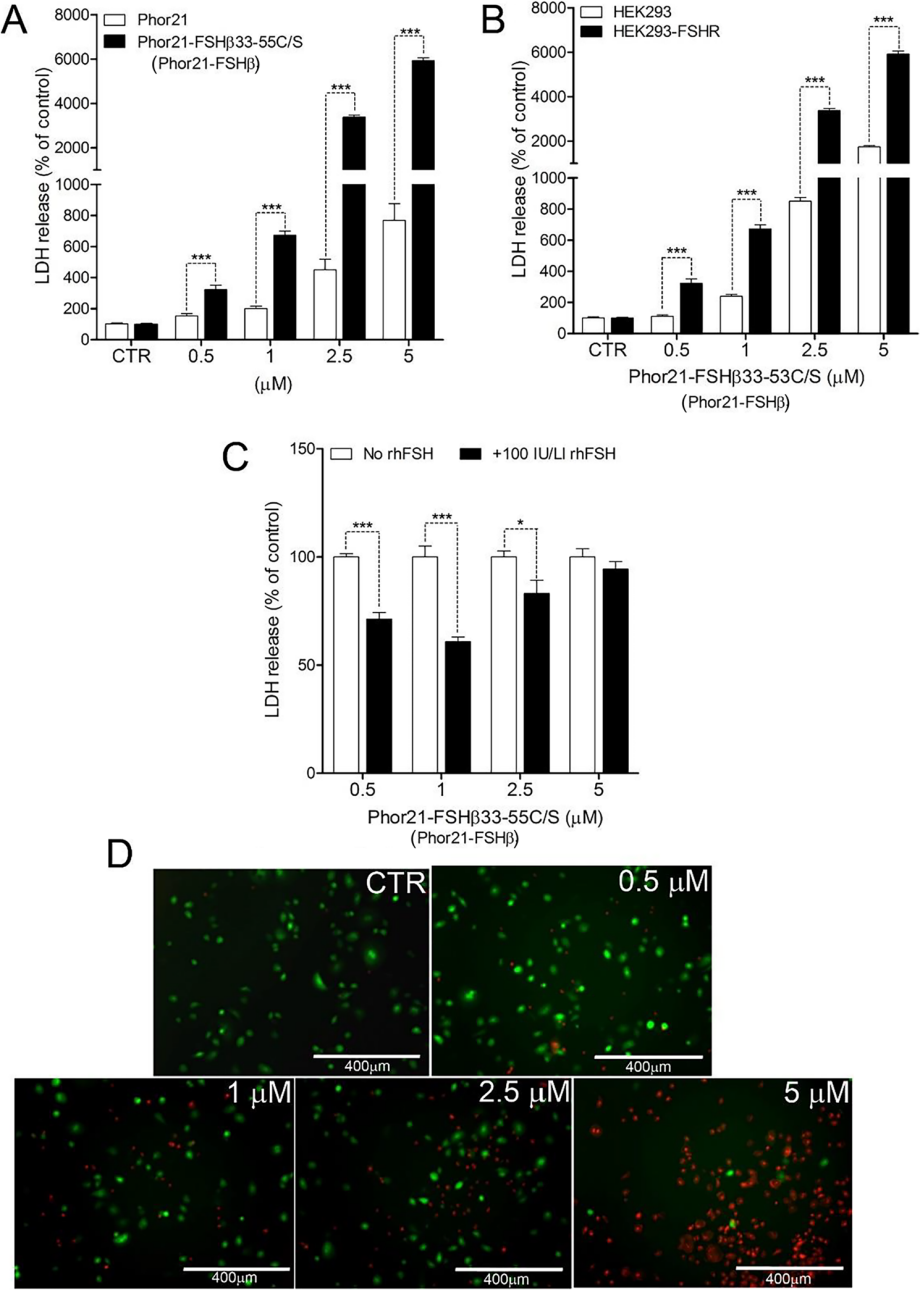
Characterization of Phor21-FSHβ33–53 C/S (further mentioned as Phor21-FSHβ) conjugate cytotoxicity and specificity in vitro determined by the release of lactose dehydrogenase (LDH) into the culture supernatant (**A**-**D**) and fluorescent probe imaging (**E**). **A** Dose-dependent Phor21-FSHβ33–53 C/S (Phor21-FSHβ) –induced cytotoxicity in HEK293-FSHR cells. **B** Comparison of Phor21 and Phor21-SHβ33–53 C/S (Phor21-FSHβ) cytotoxicity in HEK293-FSHR cells. **C** Comparison of the sensitivity of HEK293 and HEK293-FSHR cells to Phor21-FSHβ33–53 C/S (Phor21-FSHβ) cytotoxicity. **D** Effects of pre- and co-treatment with rhFSH (100 IU/L) on Phor21-FSHβ33–53 C/S (Phor21-FSHβ) -mediated cytotoxicity in HEK293-FSHR cells. The values are the means ±SEMs of three independent experiments (*n* = 8/experiment) in three different passages of the cell line. Bars with different superscript letters differ significantly from each other (*P* ≤ 0.05). Asterisks indicate significant differences (*P ≤ 0.05; ***P ≤ 0.001) between the indicated groups. (**E**) Live/dead viability test of Phor21-FSHβ33–53 C/S (Phor21-FSHβ) -treated HEK293-FSHR cells discriminating live from dead cells by simultaneous staining with green-fluorescent calcein-AM (live) and red-fluorescent propidium iodide (dead). HEK293, human embryonic kidney 293 cell line; HEK293-FSHR, HEK293 cells stably transfected with FLAG-hFSHR/pcDNA3.1; rhFSH, recombinant human follicle-stimulating hormone

Analysis of the conjugate-mediated cytotoxicity of the Phor21-FSHβ33–53 C/S (Phor21-FSHβ) conjugate, even at the two lowest doses (0.5 and 1 µM), revealed that it had the highest cytotoxicity against HEK293-FSHR cells compared with the other tested conjugates (Fig. S3). To analyze the specificity of Phor21-FSHβ in targeting FSHR-positive cells, we compared the dose-dependent cytotoxicity of the Phor21-FSHβ conjugate and the Phor21 backbone in HEK293-FSHR and HEK-293 cells. The dose-dependent cytotoxicity of the Phor21-FSHβ conjugate was significantly (up to 7.5-fold) higher than that of the Phor21 backbone (Fig. 2A), or in HEK293-FSHR cells vs. HEK-293 cells (Fig. 2B; up to 4-fold). A decrease in Phor21-FSHβ33–53 C/S-mediated cytotoxicity, at doses ranging from 0.5 to 2.5 µM, was detected in HEK293-FSHR cells pre- and co-stimulated with 100 IU/L rhFSH (Fig. 2C). This finding confirmed the specific binding of the Phor21-FSHβ conjugate to FSHR and suggested potential competition between endogenous FSH and the conjugate for FSHR. Specific cytotoxicity of the Phor21-FSHβ conjugate toward FSHR-possessing cells was also observed by the live/dead viability test (2 D).

### Phor21-FSHβ33–55 C/S (Phor21-FSHβ) specifically inhibited xenograft growth of HEK293-FSHR cells

**Fig. 3:**
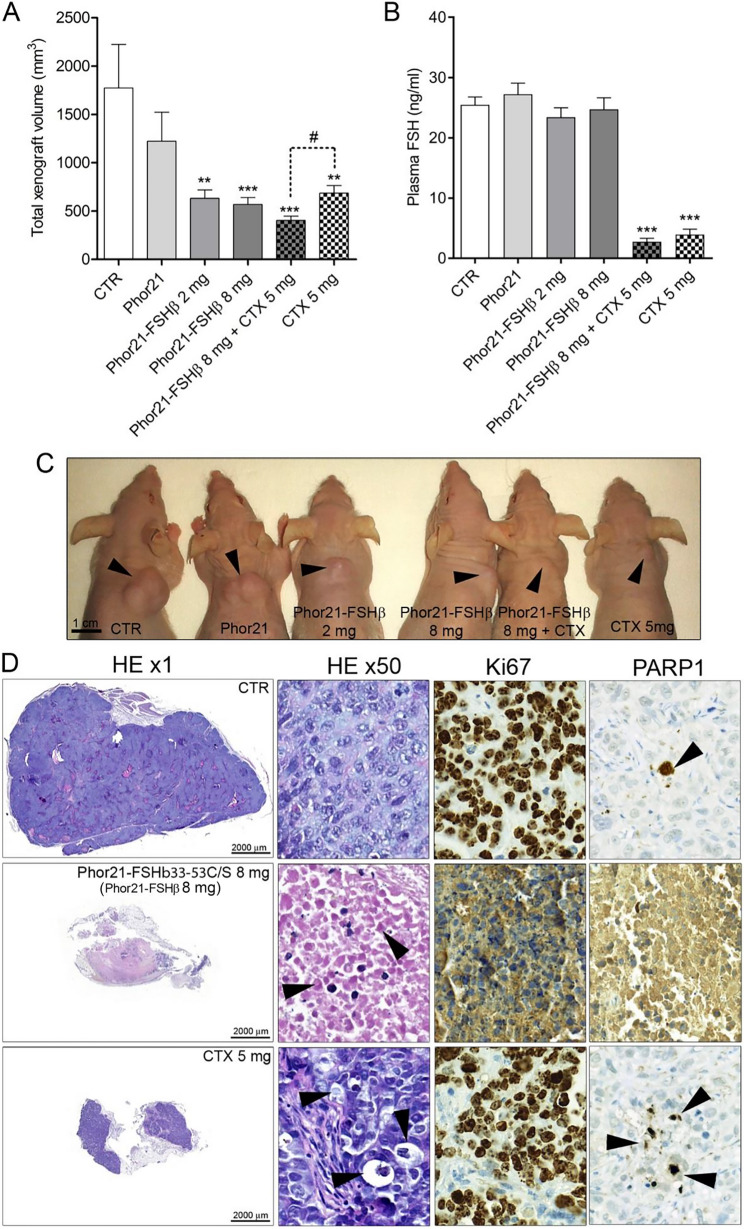
Effects of Phor21-FSHβ33–53 C/S (Phor21-FSHβ) and cetrorelix (CTX) treatments on HEK293-FSHR xenograft growth. **A** Total xenograft volume at necropsy. **B** Plasma FSH levels. **C** Representative images of HEK293-FHSR xenografts that developed subcutaneously in the interscapular area of nude mice. **D** Histology and immunohistochemical staining for nuclear Ki-67 and cytoplasmic active caspase 3/7 apoptosis markers in xenografts from control and Phor21-FSHβ or CTX-treated mice. The arrows indicated shrunken cells with condensed cytoplasm and pyknotic and fragmented nuclei. The values are the means ±SEMs (*n* = 10–12). Asterisks/hashtags indicate significant differences between the CTR group and the treatments or indicated groups (*P ≤ 0.05; ***P ≤ 0.001) Cetrorelix, CTX; CTR, control treated with vehicle; HEK293, human embryonic kidney 293 cell line; HEK293-FSHR, HEK293 cells stably transfected with the FLAG-hFSHR/pcDNA3.1 expression plasmid

In contrast to Phor21, both doses of Phor21-FSHβ, alone or in combination with CTX, inhibited xenograft growth compared with that in the control group (Fig. 3A). As expected, CTX significantly decreased FSH secretion (Fig. 3B) but also reduced xenograft growth (Fig. 3A, B). The combined effect of Phor21-FSHβ and CTX on reducing xenograft growth was significantly higher (#, *p* = 0.012) than that of CTX alone (Fig. 3A, B). Histopathological analysis of xenograft tissues revealed massive necrotic changes in all Phor21-FSHβ-treated mice (Fig. 3D). CTX treatment-induced apoptosis manifested histologically as shrunken cells with condensed cytoplasm and pyknotic and fragmented nuclei (arrowheads, Fig. 3D) and was confirmed by cytoplasmic staining of the apoptosis marker PARP1 (Fig. 3D). In contrast to the control and CTX groups, Ki67 was not expressed in the Phor21-treated xenografts (Fig. 3D).

No significant differences in body, testis, or spleen weights were detected between Phor21- and Phor21-FSHβ33–55 C/S-treated nude male xenografts with HEK293-FSHR cells (Fig. S4 A‒C). However, CTX alone or in combination with Phor21-FSHβ33–55 C/S significantly reduced the testis weight and increased the spleen size (Fig. S4 B, C). The effects of Phor21-FSH and cetrorelix (CTX) treatments on tumor growth (xenograft) (% of control) in HEK293-FSHR and LNCaP cells were significantly decreased in HEK-293-FSHR cells within the xenografts treated with 8 mg and 2 mg of Phor21-FSHβ33–55 C/S alone or 8 mg with or without 5 mg of CTX (Fig S5).

**Fig. 4:**
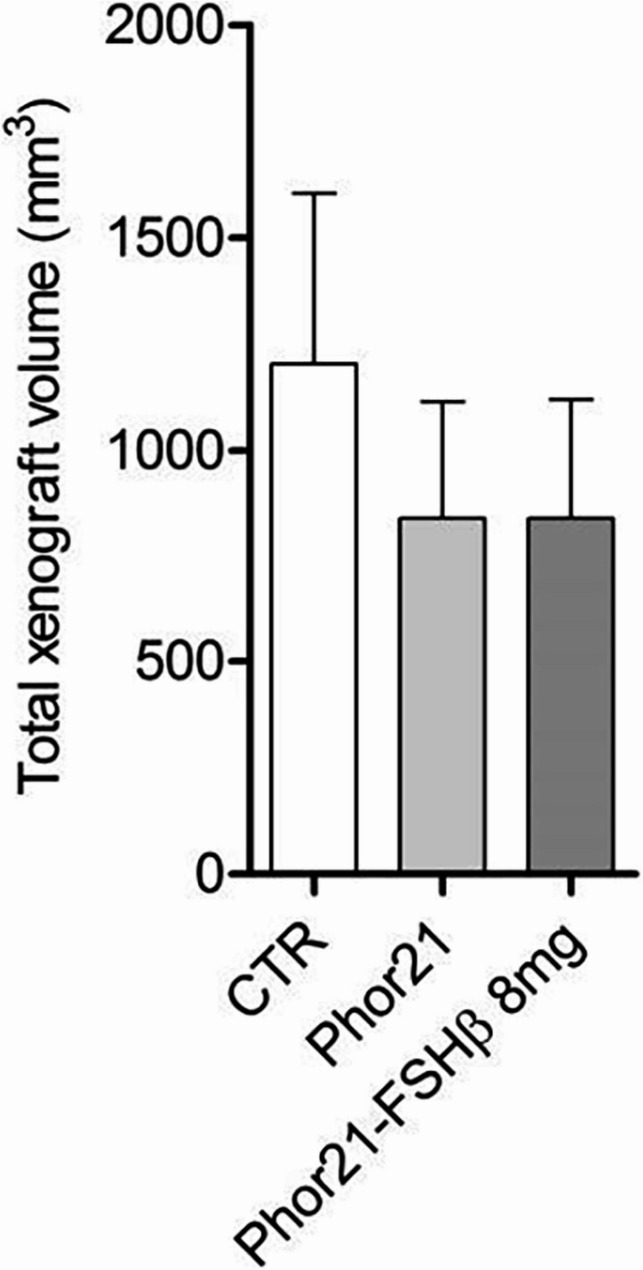
Effect of Phor21-FSHb33-53 C/S (Phor21-FSHβ) treatment on HEK-293 xenograft growth. Each bar represents the mean total xenograft volume measured by calipers at necropsy (*n* = 8–12)

The specificity of the Phor21-FSHβ33–53 C/S conjugate in vivo was further examined by treating FSHR-negative HEK-293 cell xenografts. This experiment revealed that the Phor21 backbone and/or Phor21-FSHβ did not affect the growth of the HEK-293 xenografts (Fig. 4). Dose-dependent treatment with the Phor21 backbone and Phor21-FSHβ had similar cytotoxic effects, as shown by LDH release (Fig S6). There was no difference in the levels of regarding serum enzymes such as GGT. AST and ALT between the CTR group and any other treatment group (Fig S7).

### Systemic and direct effects of CTX on HEK293-FSHR cells

**Fig. 5:**
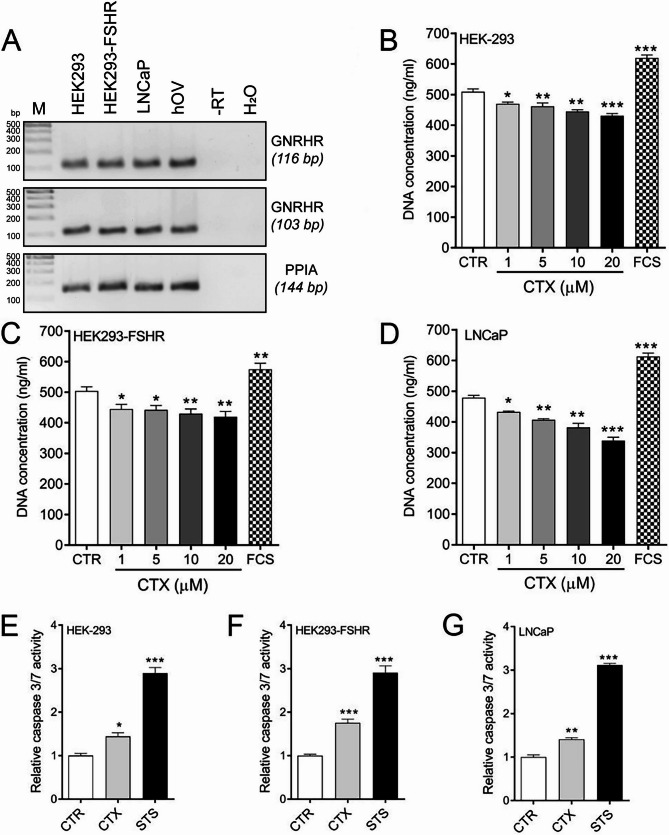
The mechanism of action of the GnRH antagonist cetrorelix (CTX). **A** *GNRHR* expression in HEK-293, HEK293-FSHR and prostate cancer LNCaP cells. **B**-**D** Effects of CTX on the proliferation of HEK-293, HEK293-FSHR and LNCaP cells. Fetal calf serum (2.5%) was used as a positive control stimulant. (E-G) Effects of CTX (20 µM) on caspase 3/7 activity. Staurosporin (STS, 0.5 µM) was used as a positive inducer of caspase3/7-mediated apoptosis. Each bar represents the mean ±SEM of three independent experiments (*n* = 8/experiment). Asterisks indicate significant differences (*P ≤ 0.05, **P ≤ 0.01, ***P ≤ 0.01) between control and stimulated cells. Cetrorelix, CTX; GnRH, gonadotropin-releasing hormone; HEK293, human embryonic kidney 293 cell line; HEK293-FSHR, HEK293 cells stably transfected with FLAG-hFSHR/pcDNA3.1; LNCaP, human prostate cancer cell line; STS, staurosporin

To determine the mechanism of CTX action on xenografts, we analyzed the mRNA expression of the GnRH receptor (*GNRHR*) in HEK-293, HEK293-FSHR, and LNCaP cells and the effects of CTX on cell proliferation and apoptosis. *GNRHR* was expressed in HEK-293, HEK293-FSHR, and LNCaP cells (Fig. 5A). CTX treatment dose-dependently decreased proliferation (Fig. 5B-D) and activated caspase3/7 (Fig. 5E-G) in all stimulated cells.

### Phor21-FSHβ33–55 C/S did not inhibit the growth of LNCaP cell xenografts—a revisit of FSHR expression in xenograft tumor vessel endothelial cells

**Fig. 6:**
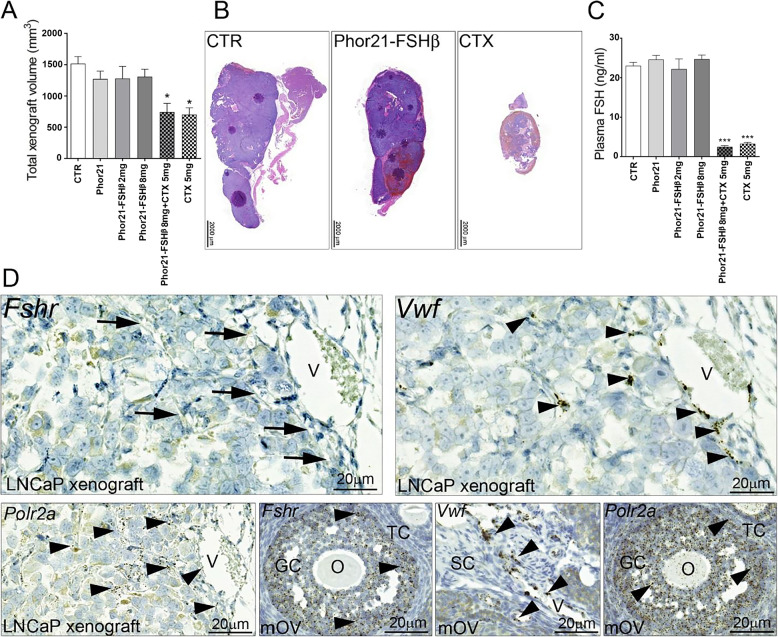
Effects of Phor21-FSHb33-53 C/S (Phor21-FSHβ) and cetrorelix (CTX) treatments on xenograft growth in LNCaP cells. **A** Total xenograft volume at necropsy. **B** Histology of LNCaP xenografts from control and Phor21-FSHb33-53 C/S (Phor21-FSHβ)- or CTX-treated mice. **C** Plasma FSH levels. The values are the means ±SEMs (*n* = 10–12). Asterisks indicate significant differences between the CTR and the treatments or indicated groups (*P ≤ 0.05; ***P ≤ 0.001). **D** RNA scope in situ hybridization of adjacent sections of LNCaP cell xenografts. Lack of *Fshr* (arrows) in *Vwf-*positive murine xenograft vessel endothelial cells (arrowheads). A mouse ovary was used as a positive control tissue for the *Fshr* and *Vwf* probes, while the *Pol2a* probe was used as a housekeeping gene (arrow heads). Cetrorelix, CTX; CTR, control treated with vehicle; GC, granulosa cells; LNCaP, human prostate cancer cell line; O, oocyte; SC, stroma cells; STS, staurosporine; TC, theca cells; V, vessel

FSHR protein and mRNA levels have been reported in tumor vessel endothelial cells of the human prostate and LNCaP xenograft vessels (of murine origin) (Radu et al. 2010). Furthermore, the proangiogenic action of FSH in HUVECs and mouse placental vessels has been demonstrated (Stilley et al. 2014a, b). These findings prompted us to examine the effects of Phor21-FSHβ33–55 C/S on FSHR-positive tumor vessel endothelial cells and LNCaP cell xenograft growth. There were no changes in the growth or histology of LNCaP xenografts treated with Phor21 or the Phor21-FSHβ33–53 C/S conjugate (2 or 8 mg) (Fig. 6A,‒C). CTX alone or in combination with Phor21-FSHβ33–53 C/S inhibited xenograft growth and inhibited FSH secretion (Fig. 6A, C). RNAscope in situ hybridization revealed the expression of von Willebrand factor (*Vwf*), but no *Fshr* transcripts were detected in the vessel endothelial cells of the LNCaP xenografts (Fig. 6D). The mouse ovary used as a positive control expressed *Fshr* in granulosa cells, and *Vwf* was found only in endothelial cells, whereas the transcripts of the housekeeping gene *Polr2a* were localized in all ovarian cells (Fig. 6D).

## Discussion

In the present study, we showed that the fusion peptide Phor21-FSHβ33–55 C/S selectively destroyed FSHR-expressing cells in vitro and prevented xenograft growth in vivo without causing any noticeable systemic toxicity. Phor21-FSHβ33–55 C/S was selected from the 12 designed conjugates because it was the most specific and cytotoxic to FSHR-positive cells at the lowest effective dose. Additionally, we revisited FSHR expression in the endothelial cells of LNCaP cell xenografts (Ghinea 2010).

In contrast to earlier studies, we were unable to show stable and functional expression of FSHR in PC-3, OVCAR-3, CaOV-3, or HEK293 cells (Yang et al. 2016; Zhang et al. 2009). Furthermore, none of these cell lines responded to rhFSH stimulation via cAMP production, which is the main second messenger involved in FSHR activation. To test the specificity and cytotoxicity of the synthesized Phor21-FSHβ conjugates, we established a versatile in vitro model stably transfected with FSHR (HEK293-FSHR) cells. HEK-293 cells [human embryonic kidney cell lines transfected with HAdV type 5 (AD5) DNA] present several features that classify them as tumorigenic cell lines, such as a negatively charged membrane, unlimited division, and stable growth when xenografted in nude mice (Stepanenko and Dmitrenko 2015). Moreover, they are suitable models because of their nondemanding culture conditions and are amenable to simple and efficient genetic manipulation (Stepanenko and Dmitrenko 2015). The greatest advantage of the HEK-293/HEK293-FSHR model was that FSHR expression was constitutive in them.

The FSHβ chain contains two specific fragments (33–53 and 81–96) responsible for binding to FSHR, which were confirmed to be effective antagonists of FSHR activation (Santa Coloma and Reichert 1990; Santa-Coloma et al. 1991). Both the FSHβ fragments and their combination (FSHβ33–53/81–96) were conjugated with the lytic peptide Phor21 to target FSHR-expressing cancer cells. Cysteine (Cys), which is present in selected FSHβ fragments, is susceptible to rapid oxidation or disulfide linkage formation. Rapid oxidation or the formation of disulfide linkages may negatively influence synthesis, subsequent peptide purification, and stability during experiments (Giles et al. 2003). Therefore, we modified these AAs by replacing them with serine (Ser) and alanine (Ala) or stabilizing them with an acetamidomethyl group (Acm). A previous study with modified synthetic analogs of hFSHβ showed that the replacement of Cys with Ser did not affect receptor-binding affinity, although it was deleterious to agonist activity (Santa-Coloma et al. 1992; Grasso et al. 1993). In our study, the replacement of cysteine residue 51 in the Phor21-FSHβ33–55 C/S conjugate (in comparison with other modifications) significantly increased its cytotoxicity, probably due to its increased stability. A similar effect was observed in the Phor21-βCG(Ala) conjugate, where cysteines in the βCG81–96 sequence were replaced with alanine (Jia et al. 2008; Hansel et al. 2007a, b) or, similar to the Hecate-CG conjugate, with cysteines stabilized by Acm groups. Phor21-βCG(Ala) treatment is more effective in reducing primary tumor weight in nude mice xenografted with human prostate and breast cancer cells or metastatic cells in the bones and lymph nodes (Jia et al. 2008; Hansel et al. 2007a, b).

The cytotoxicity of the lytic peptide Phor21 or its shorter version Phor14 has been successfully tested in combination with βCG (Phor21-βCG) to target LHCGR-positive breast (Hansel et al. 2007a, b), prostate (Leuschner et al. 2003) and ovarian (Gawronska et al. 2002) cancer cells in vitro and in xenografts. A novel variant of the lytic peptide phor, composed of 18 amino acids (Phor18) conjugated to FSHβ has been shown to inhibit the growth of PC-3 xenografts by targeting FSHR-expressing cancer or endothelial cells of tumor vessels (Aggarwal et al. 2015). The FSHβ33–53 fragment has also been fused with the cationic peptide G(IIKK)_3_I-NH_2_ (FSHR_33−53_-IIKK), which shows strong cytotoxicity against different cell lines, which, according to the authors, expresses functional FSHR (Yang et al. 2016). However, in the two above-mentioned studies, neither *FSHR* mRNA expression nor functionality was observed in these cell lines (Yang et al. 2016; Aggarwal et al. 2015). This raises concerns about the unproven specificity of the action of FSHR (Yang et al. 2016; Aggarwal et al. 2015). In our study, we found a similar dose-dependent cytotoxic effect of the Pho21 backbone and its conjugate, Phor21-FSHβ33–55 C/S, in HEK-293 cells that did not express FSHR. Therefore, to exclude nonspecific effects, efficacy studies of receptor-targeted drug treatments should be supported by target receptor mRNA, protein, and functional analyses.

In vitro studies revealed that rhFSH co-treatment reduced the efficacy of Phor21-FSHβ33–55 C/S-mediated cytotoxicity in FSHR-positive cells, indicating competition between the therapeutic compound and endogenous FSH. To avoid this competition during in vivo treatment, we combined Phor21-FSHβ33–55 C/S with CTX to block the release of FSH and luteinizing hormone (LH) from the pituitary (Kumar and Sharma 2014). The stronger inhibition of xenograft growth after combined Phor21-FSHβ33–55 C/S and CTX treatment may involve the following mechanisms. First, CTX-mediated elimination of circulating FSH increased the binding of Phor21-FSHβ33–55 C/S to FSHR and the destruction of HEK293-FSHR cells. Second, CTX treatment, independent of Phor21-FSHβ33–55 C/S, directly affected HEK293-FSHR cells. CTX has been shown to have direct antiproliferative and apoptotic effects on a number of human cancers, including prostate, colorectal, endometrial, lung, and ovarian cancers (Limonta et al. 2012). In our in vitro studies, CTX decreased proliferation and activated caspase 3/7 in HEK-293, HEK293-FSHR, and LNCaP cells. A third possibility is that xenograft growth is stimulated by circulating FSH.

The rapid cell death (< 1.5 h) observed in vitro suggested that Phor21-FSHβ33–55 C/S, like other lytic peptide-based conjugates, induced necrosis rather than apoptosis (Bodek et al. 2005a, b). Histological signs of necrosis were also observed in HEK293-FSHR cell xenografts treated with Phor21-FSHβ33–55 C/S. In contrast to chemically cytotoxic compounds, lytic peptides perforate the cell membrane and induce cell swelling and bursting, resulting in rapid cell death (Bodek et al. 2005a, b). It is well known that the serum levels of ALT, AST and GGT enzymes, which are markers of liver damage, are significantly increased (Zhong et al. 2017; Kwo et al. 2017). In the present study, Phor21-FSHβ33–55 C/S, as well as CTX treatments in vivo did not affect the ALT, AST and GGT enzymes, which suggested their treatment efficacy without inducing liver toxicity.

Unlike Radu et al. (2010), we were unable to determine *Fshr* expression in LNCaP xenograft vasculature in mice. We used the sensitive commercial RNAScope in situ hybridization method, which allows visualization of single *FSHR* transcripts. However, we cannot rule out technical details as the cause of the discrepancy between our findings and those of the other studies. Previously published data were based on immunohistochemistry using FSHR323 monoclonal antibody (Radu et al. 2010) or unknown (Yang et al. 2016; Zhang et al. 2009) antibodies against FSHR, usually without any additional methodological confirmation. In most of the studies, no control samples (positive or negative) were included to validate the specificity of the antibodies (Yang et al. 2016; Zhang et al. 2009). Even when tissue controls were used, a high background of FSHR-negative cells/tissues was observed (Stilley et al. 2014a, b). Moreover, the high FSHR IHC or IF immunoreactivity observed in other studies was not followed by high *FSHR* mRNA expression, suggesting nonspecific signal in antibody-based data (Chrusciel et al. 2019).

## Conclusions

**Fig. 7:**
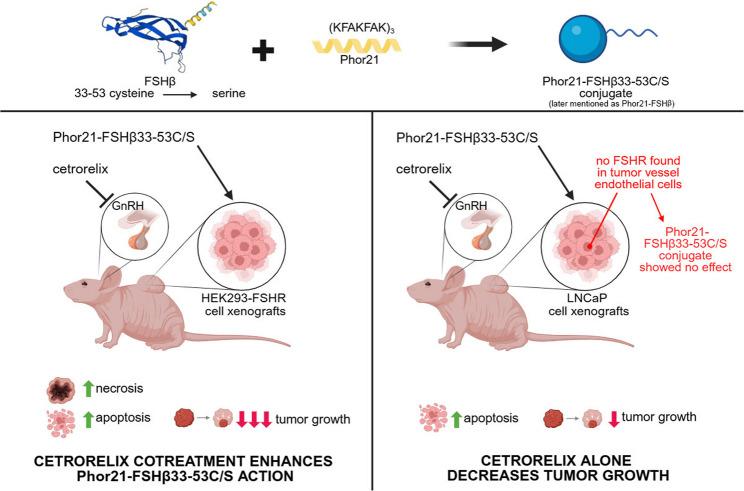
Graphical Abstract Schematic overview of the Phor21-FSHb33-53 C/S (Phor21-FSHβ) conjugate or CTX specifically targeted to kill FSHR-positive cancer cells. (Figure created using BioRender.com). Phor21-FSHb33-53 C/S conjugate, Phor21 lytic backbone conjugated with a native or modified fragment of the FSHβ subunit (FSHβ33–53); CTX, GnRH antagonist cetrorelix

Taken together, our findings indicate that the Phor21-FSHβ33–55 C/S conjugate had a strong cytotoxic effect on cancer cells expressing FSHR. A schematic overview of the ability of the Phor21-FSHβ33–55 C/S conjugate or CTX to selectively ablate the FSHR-positive cancer cells is shown in Fig. 7. The high selectivity, apparent proteolytic stability, and low systemic toxicity of Phor21-FSHβ33–55 C/S suggest considerably high therapeutic potential. The combination of Phor21-FSHβ33–55 C/S treatment with a GnRH antagonist is recommended to eliminate competition between the conjugate and FSH for binding to the FSHR expressed in cancer cells. The negative results of *Fshr mRNA* expression in the endothelial cells of LNCaP xenograft murine vessels, supported by the negative expression of FSHR in HUVECs (Rivero-Muller et al. 2007), indicate that FSHR expression in the prostate cancer vasculature (Radu et al. 2010), as a potential therapeutic target, requires further confirmation.

## Supplementary Information

Supplementary Material 1.

## Data Availability

No datasets were generated or analysed during the current study.

## Abbreviations

CANDLES: Cyclic AMP iNdirect detection by light emission from sensor cell assay
FLAG: Polypeptide protein tag
FSHR: Follicle-stimulating hormone receptor
HEK293: Human embryonic kidney 293 cell line
HEK293-FSHR: HEK293 cells stably transfected with FLAG-hFSHR/pcDNA3.1
OV: Ovary
rhFSH: recombinant human follicle-stimulating hormone
cetrorelix: CTX (gonadotropin-releasing hormone antagonist)
CTR: Control treated with vehicle
GnRH: Gonadotropin-releasing hormone
LNCaP: Human prostate cancer cell line
STS: Staurosporin
GC: Granulosa cells
O: Oocyte
SC: Stroma cells
TC: Theca cells
V: Vessel
FCS: Fetal calf serum
FRK: Forskolin

## References

[CR1] Aggarwal SGT, Alila H, Leuschner C, Karki N, Solipuram R, Wang Q, Hansel W. Anti-tumor effects of targeted follicle-stimulating hormone-lytic peptide conjugates in prostate cancer (PC-3) xenograft mouse model. Int J Cancer Res Mol Mech. 2015;1:12–20

[CR2] Ben-Josef E, et al. Hormone-refractory prostate cancer cells express functional follicle-stimulating hormone receptor (FSHR). J Urol. 1999;161:970–6. 10022736

[CR3] Bodek G, et al. A novel approach of targeted ablation of mammary carcinoma cells through luteinizing hormone receptors using Hecate-CGbeta conjugate. Breast Cancer Res Treat. 2003;79:1–10. 12779076 10.1023/a:1023351819956

[CR4] Bodek G, Kowalczyk A, Waclawik A, Huhtaniemi I, Ziecik AJ. Targeted ablation of prostate carcinoma cells through LH receptor using Hecate-CGbeta conjugate: functional characteristic and molecular mechanism of cell death pathway. Exp Biol Med (Maywood). 2005a;230:421–8. 15956772 10.1177/15353702-0323006-10

[CR5] Bodek G, et al. A novel targeted therapy of Leydig and granulosa cell tumors through the luteinizing hormone receptor using a hecate-chorionic gonadotropin beta conjugate in Transgenic mice. Neoplasia. 2005b;7:497–508. 15967102 10.1593/neo.04751 PMC1501163

[CR6] Bogacki M, Enright FM, Todd WJ, Hansel W. Immune response to lytic peptides conjugated to a BetaCG fragment in treated BALB/C mice. Reprod Biol. 2008;8:135–47. 18677401 10.1016/s1642-431x(12)60009-6

[CR7] Bose CK. Follicle stimulating hormone receptor in ovarian surface epithelium and epithelial ovarian cancer. Oncol Res. 2008;17:231–8. 18980020 10.3727/096504008786111383

[CR8] Casarini L, et al. Membrane estrogen receptor (GPER) and follicle-Stimulating hormone receptor (FSHR) heteromeric complexes promote human ovarian follicle survival. iScience. 2020;23:101812. 33299978 10.1016/j.isci.2020.101812 PMC7702187

[CR9] Choi JH, Choi KC, Auersperg N, Leung PC. Overexpression of follicle-stimulating hormone receptor activates oncogenic pathways in preneoplastic ovarian surface epithelial cells. J Clin Endocrinol Metab. 2004;89:5508–16. 15531506 10.1210/jc.2004-0044

[CR10] Choong CS, et al. Sertoli-Leydig cell tumor of the ovary, a rare cause of precocious puberty in a 12-month-old infant. J Clin Endocrinol Metab. 2002;87:49–56. 11788622 10.1210/jcem.87.1.8162

[CR11] Chrusciel M, Ponikwicka-Tyszko D, Wolczynski S, Huhtaniemi I, Rahman NA. Extragonadal FSHR expression and Function-Is it real?? Front Endocrinol (Lausanne). 2019;10:32. 30778333 10.3389/fendo.2019.00032 PMC6369633

[CR12] Chu S, et al. FSH-regulated gene expression profiles in ovarian tumours and normal ovaries. Mol Hum Reprod. 2002;8:426–33. 11994539 10.1093/molehr/8.5.426

[CR13] Gawronska B, Leuschner C, Enright FM, Hansel W. Effects of a lytic peptide conjugated to beta HCG on ovarian cancer: studies in vitro and in vivo. Gynecol Oncol. 2002;85:45–52. 11925119 10.1006/gyno.2001.6558

[CR14] Ghinea N. A novel role for FSH receptor as a tumor endothelial cell marker. Acta Endocrinol (Bucharest). 2010;6:507–12.

[CR15] Giles NM, Giles GI, Jacob C. Multiple roles of cysteine in biocatalysis. Biochem Biophys Res Commun. 2003;300:1–4. 12480511 10.1016/s0006-291x(02)02770-5

[CR16] Grasso P, Crabb JW, Reichert LE Jr. An explanation for the disparate effects of synthetic peptides corresponding to human follicle-stimulating hormone beta-subunit receptor binding regions (33–53) and (81–95) and their Serine analogs on steroidogenesis in cultured rat Sertoli cells. Biochem Biophys Res Commun. 1993;190:56–62. 8422260 10.1006/bbrc.1993.1010

[CR17] Griswold MD, Heckert L, Linder C. The molecular biology of the FSH receptor. J Steroid Biochem Mol Biol. 1995;53:215–8. 7626457 10.1016/0960-0760(95)00049-6

[CR18] Hansel W, Leuschner C, Gawronska B, Enright F. Targeted destruction of prostate cancer cells and xenografts by lytic peptide-betaLH conjugates. Reprod Biol. 2001;1:20–32. 14666172

[CR19] Hansel W, Leuschner C, Enright F. Conjugates of lytic peptides and LHRH or BetaCG target and cause necrosis of prostate cancers and metastases. Mol Cell Endocrinol. 2007a;269:26–33. 17382461 10.1016/j.mce.2006.06.017

[CR20] Hansel W, Enright F, Leuschner C. Destruction of breast cancers and their metastases by lytic peptide conjugates in vitro and in vivo. Mol Cell Endocrinol. 2007b;260–262:183–9. 17101210 10.1016/j.mce.2005.12.056

[CR21] Harper JF, Brooker G. Femtomole sensitive radioimmunoassay for Cyclic AMP and Cyclic GMP after 2’0 acetylation by acetic anhydride in aqueous solution. J Cycl Nucleotide Res. 1975;1:207–18. 177461

[CR22] Jia L, et al. Pharmacokinetics and pharmacodynamics of Phor21-betaCG(ala), a lytic peptide conjugate. J Pharm Pharmacol. 2008;60:1441–8. 18957164 10.1211/jpp.60.11.0004 PMC2825277

[CR23] Jonas KC, et al. Temporal reprogramming of calcium signalling via crosstalk of gonadotrophin receptors that associate as functionally asymmetric heteromers. Sci Rep. 2018;8:2239. 29396488 10.1038/s41598-018-20722-5 PMC5797151

[CR24] Kumar P, Sharma A. Gonadotropin-releasing hormone analogs: understanding advantages and limitations. J Hum Reprod Sci. 2014;7:170–4. 25395741 10.4103/0974-1208.142476 PMC4229791

[CR25] Kwo PY, Cohen SM, Lim JK. ACG clinical guideline: evaluation of abnormal liver chemistries. Am J Gastroenterol. 2017;112:18–35. 27995906 10.1038/ajg.2016.517

[CR26] Lei ZX, Wang JJ, Li K, Liu P. Herp knockout protects against nonalcoholic fatty liver disease in mice on a high fat diet. Kaohsiung J Med Sci. 2021;37:487–96. 33464700 10.1002/kjm2.12349 PMC11896413

[CR27] Leuschner C, Hansel W. Membrane disrupting lytic peptides for cancer treatments. Curr Pharm Des. 2004;10:2299–310. 15279610 10.2174/1381612043383971

[CR28] Leuschner C, Enright FM, Gawronska-Kozak B, Hansel W. Human prostate cancer cells and xenografts are targeted and destroyed through luteinizing hormone releasing hormone receptors. Prostate. 2003;56:239–49. 12858351 10.1002/pros.10259

[CR29] Limonta P, et al. GnRH receptors in cancer: from cell biology to novel targeted therapeutic strategies. Endocr Rev. 2012;33:784–811. 22778172 10.1210/er.2012-1014

[CR30] Liu XM, et al. FSH regulates fat accumulation and redistribution in aging through the Galphai/Ca(2+)/CREB pathway. Aging Cell. 2015;14:409–20. 25754247 10.1111/acel.12331 PMC4406670

[CR31] Liu P, et al. Blocking FSH induces thermogenic adipose tissue and reduces body fat. Nature. 2017;546:107–12. 28538730 10.1038/nature22342 PMC5651981

[CR32] Mariani S, et al. Expression and cellular localization of follicle-stimulating hormone receptor in normal human prostate, benign prostatic hyperplasia and prostate cancer. J Urol. 2006;175:2072–7. discussion 2077. 16697805 10.1016/S0022-5347(06)00273-4

[CR33] Oduwole OO, et al. Follicle-stimulating hormone promotes growth of human prostate cancer cell line-derived tumor xenografts. FASEB J. 2021;35:e21464. 33724574 10.1096/fj.202002168RR

[CR34] Planeix F, et al. Endothelial follicle-stimulating hormone receptor expression in invasive breast cancer and vascular remodeling at tumor periphery. J Exp Clin Cancer Res. 2015;34:12. 25652007 10.1186/s13046-015-0128-7 PMC4321709

[CR35] Ponikwicka-Tyszko D, et al. Functional expression of FSH receptor in endometriotic lesions. J Clin Endocrinol Metab. 2016;101:2905–14. 27224263 10.1210/jc.2016-1014

[CR36] Radu A, et al. Expression of follicle-stimulating hormone receptor in tumor blood vessels. N Engl J Med. 2010;363:1621–30. 20961245 10.1056/NEJMoa1001283

[CR37] Rivero-Muller A, et al. Use of hecate-chorionic gonadotropin beta conjugate in therapy of lutenizing hormone receptor expressing gonadal somatic cell tumors. Mol Cell Endocrinol. 2007;269:17–25. 17363137 10.1016/j.mce.2006.11.016

[CR38] Santa Coloma TA, Reichert LE Jr. Identification of a follicle-stimulating hormone receptor-binding region in hFSH-beta-(81–95) using synthetic peptides. J Biol Chem. 1990;265:5037–42. 1690733

[CR39] Santa-Coloma TA, Crabb JW, Reichert LE Jr. A synthetic peptide encompassing two discontinuous regions of hFSH-beta subunit mimics the receptor binding surface of the hormone. Mol Cell Endocrinol. 1991;78:197–204. 1778304 10.1016/0303-7207(91)90123-a

[CR40] Santa-Coloma TA, Crabb JW, Reichert LE Jr. Serine analogues of hFSH-beta-(33–53) and hFSH-beta-(81–95) inhibit hFSH binding to receptor. Biochem Biophys Res Commun. 1992;184:1273–9. 1590789 10.1016/s0006-291x(05)80020-8

[CR41] Sheng S et al. Follicle-stimulating hormone promotes the development of endometrial Cancer in vitro and in vivo. Int J Environ Res Public Health. 2022;19:15344–55. 10.3390/ijerph192215344 PMC9696221 36430063

[CR42] Siraj MA, Pichon C, Radu A, Ghinea N. Endothelial follicle stimulating hormone receptor in primary kidney cancer correlates with subsequent response to Sunitinib. J Cell Mol Med. 2012;16:2010–6. 22129368 10.1111/j.1582-4934.2011.01495.x PMC3822971

[CR43] Siraj A, et al. Expression of follicle-stimulating hormone receptor by the vascular endothelium in tumor metastases. BMC Cancer. 2013;13:246. 23688201 10.1186/1471-2407-13-246 PMC3663659

[CR44] Starzynski D et al. Pilot study: FSHR expression in neuroendocrine tumors of the appendix. J Clin Med. 2023;12:5086–100. 10.3390/jcm12155086 PMC10419379 37568488

[CR45] Stelmaszewska J, et al. Revisiting the expression and function of follicle-stimulation hormone receptor in human umbilical vein endothelial cells. Sci Rep. 2016;6:37095. 27848975 10.1038/srep37095 PMC5111068

[CR46] Stepanenko AA, Dmitrenko VV. HEK293 in cell biology and cancer research: phenotype, karyotype, tumorigenicity, and stress-induced genome-phenotype evolution. Gene. 2015;569:182–90. 26026906 10.1016/j.gene.2015.05.065

[CR47] Stilley JA, Guan R, Duffy DM, Segaloff DL. Signaling through FSH receptors on human umbilical vein endothelial cells promotes angiogenesis. J Clin Endocrinol Metab. 2014a;99:E813–820. 24527712 10.1210/jc.2013-3186 PMC4010687

[CR48] Stilley JA, et al. FSH receptor (FSHR) expression in human extragonadal reproductive tissues and the developing placenta, and the impact of its deletion on pregnancy in mice. Biol Reprod. 2014b;91:74. 25100706 10.1095/biolreprod.114.118562 PMC4435062

[CR49] Trehan A, Rotgers E, Coffey ET, Huhtaniemi I, Rivero-Muller A. CANDLES, an assay for monitoring GPCR induced cAMP generation in cell cultures. Cell Commun Signal. 2014;12:70. 25366423 10.1186/s12964-014-0070-x PMC4228090

[CR50] van Casteren JI, Schoonen WG, Kloosterboer HJ. Development of time-resolved Immunofluorometric assays for rat follicle-stimulating hormone and luteinizing hormone and application on Sera of cycling rats. Biol Reprod. 2000;62:886–94. 10727257 10.1095/biolreprod62.4.886

[CR51] Wang J, et al. Quantitative analysis of follicle-stimulating hormone receptor in ovarian epithelial tumors: a novel approach to explain the field effect of ovarian cancer development in secondary mullerian systems. Int J Cancer. 2003;103:328–34. 12471615 10.1002/ijc.10848

[CR52] Wang F, et al. RNAscope: a novel in situ RNA analysis platform for formalin-fixed, paraffin-embedded tissues. J Mol Diagn. 2012;14:22–9. 22166544 10.1016/j.jmoldx.2011.08.002 PMC3338343

[CR53] Yang R, et al. An investigation on a novel anti-tumor fusion peptide of FSH33-53-IIKK. J Cancer. 2016;7:1010–9. 27313792 10.7150/jca.14425 PMC4910594

[CR54] Zhang XY, et al. Follicle-stimulating hormone peptide can facilitate paclitaxel nanoparticles to target ovarian carcinoma in vivo. Cancer Res. 2009;69:6506–14. 19638590 10.1158/0008-5472.CAN-08-4721

[CR55] Zhong S, et al. The therapeutic effect of Silymarin in the treatment of nonalcoholic fatty disease: a meta-analysis (PRISMA) of randomized control trials. Med (Baltim). 2017;96:e9061. 10.1097/MD.0000000000009061 PMC5728929 29245314

