# Supplementary material for: Targeted destruction of follicle stimulating hormone receptor-positive cancer cells in vitro and in vivo by a lytic peptide Phor21-FSHβ conjugate
Source: Mol Med. 2025 Jun 9;31:224. doi: 10.1186/s10020-025-01292-5 (PMC12147355; doi:10.1186/s10020-025-01292-5)

Supplementary material

Targeted destruction of folliclestimulating hormone receptor-positive cancer cells in vitro and in vivo by a lytic peptide Phor21-FSHβ conjugate

Nafis A Rahman, Marcin Chrusciel, Donata Ponikwicka-Tyszko, Kamila Pulawska-Moon, Milena Doroszko, Joanna Stelmaszewska, Oliver J Keuzer, Adolfo Rivero-Muller, Piotr Bernaczyk, Grzegorz Zalewski, Peilan Guo, Jorma Toppari, Xiangdong Li, Adam J Ziecik, Slawomir Wolczynski, Ilpo Huhtaniemi

*Corresponding author's contact information: nafis.rahman@utu.fi

Supplementary Table 1. Sequences and modifications of Phor21-FSHβ conjugates.

No.	Name	Peptide sequence	MW (Da) Found/Calc.		Purity (%)
1.	Phor21	KFAKFAKKFAKFAKKFAKFAK-CONH2	2484.7	2481.1	98.1
2.	Phor21-FSHβ (33-53)	KFAKFAKKFAKFAKKFAKFAK-YTRDLVYKDPARPKIQKTCTF-CONH2	5012.0	5007.0	95.7
3.	Phor21-FSHβ (81-95)	KFAKFAKKFAKFAKKFAKFAK-QCHCGKCDSDSTDCT-CONH2	4067.1	4065.8	95.5
4.	Phor21-FSHβ (33-53+81-95)	KFAKFAKKFAKFAKKFAKFAK-YTRDLVYKDPARPKIQKTCTF -QCHCGKCDSDSTDCT-CONH2	6596.2	6591.7	93.8
5.	Phor21-FSHβ (33-53, C/S)	KFAKFAKKFAKFAKKFAKFAK-YTRDLVYKDPARPKIQKT**S**TF-CONH2	4991.3	4990.1	98.9
6.	Phor21-FSHβ (81-95, C/S)	KFAKFAKKFAKFAKKFAKFAK-Q**S**H**S**GK**S**DSDSTD**S**T-CONH2	4000.9	4000.6	95.7
7.	Phor21-FSHβ (C/S)	KFAKFAKKFAKFAKKFAKFAK-YTRDLVYKDPARPKIQKT**S**TF - Q**S**H**S**GK**S**DSDSTD**S**T-CONH2	6511.9	6510.5	96.5
8.	Phor21-FSHβ (33-53, C/A)	KFAKFAKKFAKFAKKFAKFAK-YTRDLVYKDPARPKIQKT**A**TF-CONH2	4974.7	4974.1	98.9
9.	Phor21-FSHβ (81-95, C/A)	KFAKFAKKFAKFAKKFAKFAK-Q**A**H**A**GK**A**DSDSTD**A**T-CONH2	3937.2	3936.6	95.6
10.	Phor21-FSHβ (33-53+81-95, C/A)	KFAKFAKKFAKFAKKFAKFAK-YTRDLVYKDPARPKIQKT**A**TF - Q**A**H**A**GK**A**DSDSTD**A**T-CONH2	6431.4	6430.5	97.7
11.	Phor21-FSHβ (33-53, C-Acm)	KFAKFAKKFAKFAKKFAKFAK -YTRDLVYKDPARPKIQKT**C(Acm)**TF-CONH2	5078.2	5078.9	98.5
12.	Phor21-FSHβ (81-95, C-Acm)	KFAKFAKKFAKFAKKFAKFAK -Q**C(Acm)**H**C(Acm)**GK**C(Acm)**DSDSTD**C(Acm)**T-CONH2	4349.6	4349.0	97.8
13.	Phor21-FSHβ (33-53+81-95, C-Acm)	KFAKFAKKFAKFAKKFAKFAK- YTRDLVYKDPARPKIQKTC(Acm)TF - Q**C(Acm)**H**C(Acm)**GK**C(Acm)**DSDST D**C(Acm)**T -CONH2	6947.5	6945.9	95.3

MW – molecular weight; Acm - acetamidomethyl (C_3H_5NO) protecting group MW=72.1

Supplementary Table 2. Experiments with HEK293-FSHR, HEK-293 and LNCaP xenografts.

Experiments / Groups	EXPERIMENT 1 Cells - HEK293-FSHR 1×10^6 cells/0.2 ml Duration – 21days	EXPERIMENT 2 Cells - HEK-293 1×10^6 cells/0.2 ml Duration – 21days	EXPERIMENT 3 Cells – LNCaP 5×10^6 cells/0.2 ml Duration – 21days
CTR (vehicle)	x (n=12)	x (n=9)	x (n=12)
Phor21 8 mg/kg/72 h	x (n=10)	x (n=8)	x (n=10)
Phor21-FSHβ33-53C/S 2 mg/kg/72 h	x (n=11)		x (n=10)
Phor21-FSHβ33-53C/S 8 mg/kg/72 h	x (n=11)	x (n=10)	x (n=10)
Phor21-FSHβ33-53C/S 8 mg/kg/72 h + CTX 5 mg/kg/48h	x (n=12)		x (n=11)
CTX 5 mg/kg/48h	x (n=10)		x (n=10)

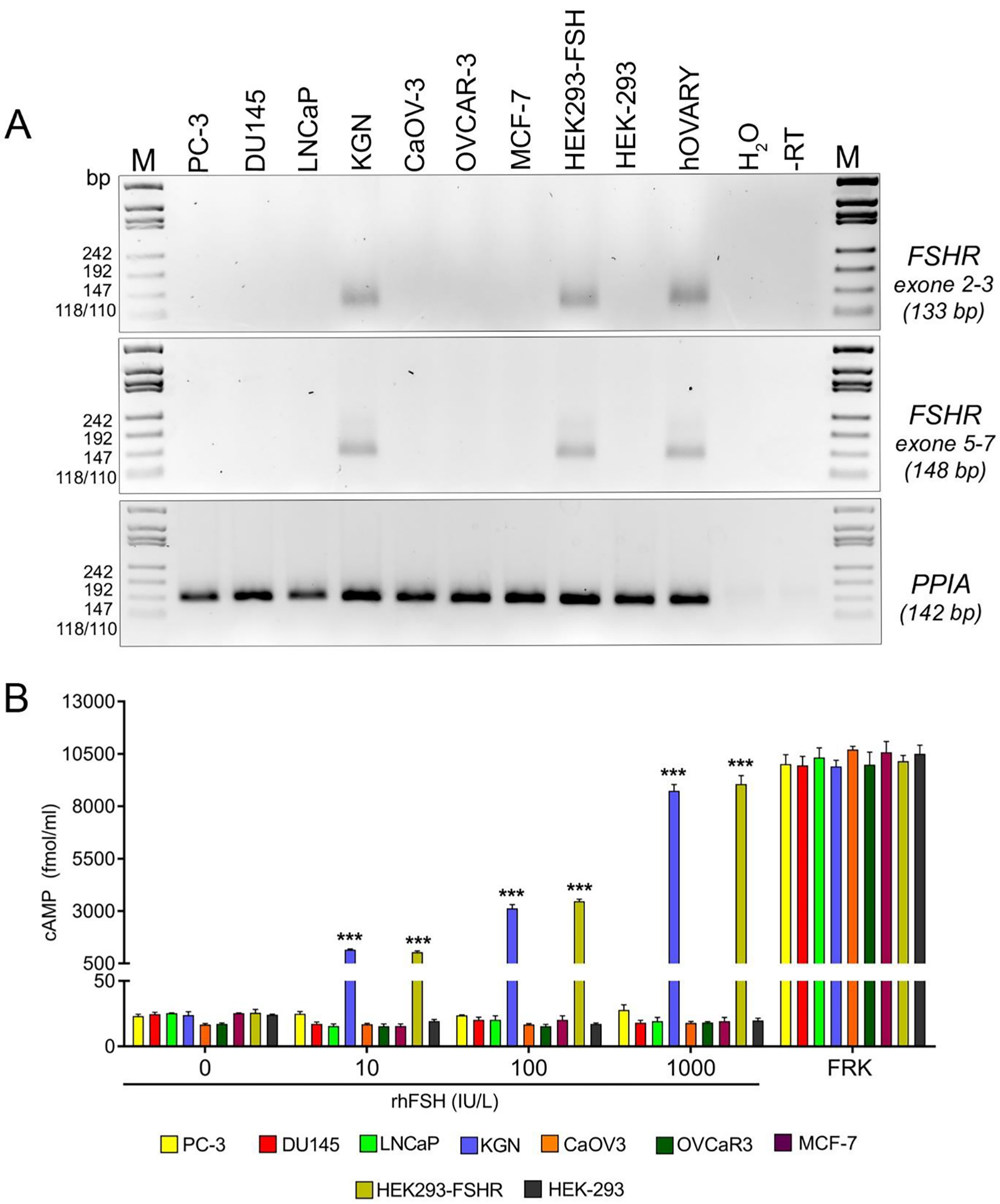

Fig. S1.

Fig. S1. Expression of functional FSHR in PC-3, DU145, LNCaP, KGN, CaOV-3, OVCAR-3, MCF-7, HEK293-FSHR and HEK-293 cells. (A) *FSHR* expression was analyzed with 2 primers spanning different exons (2-3 and 5-7) of the gene. The human ovary was used as a positive control, whereas a no template control (H_2O) and no reverse transcriptase control (-RT) were used as negative controls. Peptidylprolyl isomerase A (*PPIA*) was used as a housekeeping gene.

(B) rhFSH stimulated extracellular cAMP production.

cAMP was measured in media collected from the above-listed cells after 1 h of incubation without or with 10, 100, or 1000 IU/L or 10 μM forskolin, which was used as a positive control. Each bar represents the mean ± SEM of three independent experiments with n = 4 per treatment. Asterisks indicate differences between control and stimulated cells (***P≤0.001).

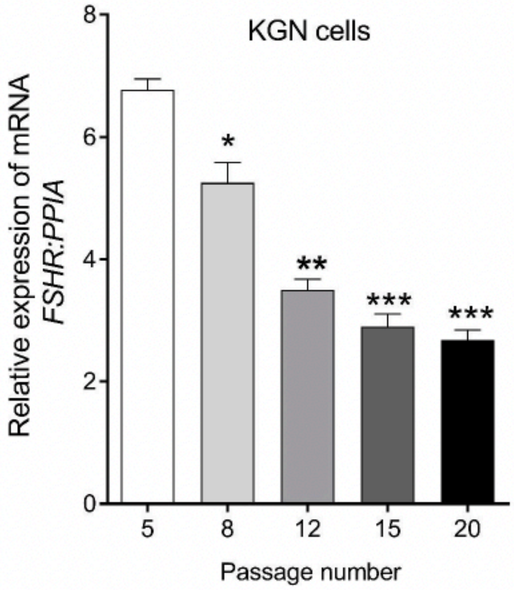

Fig. S2.

Fig. S2. Effect of passage number on the expression of *FSHR* in KGN cells. Each bar represents the mean ± SEM obtained from triplicates of analyzed cell line passages. Asterisks indicate differences between passages, with the 5th used as a control (*P≤0.05, **P≤0.01, ***P≤0.01).

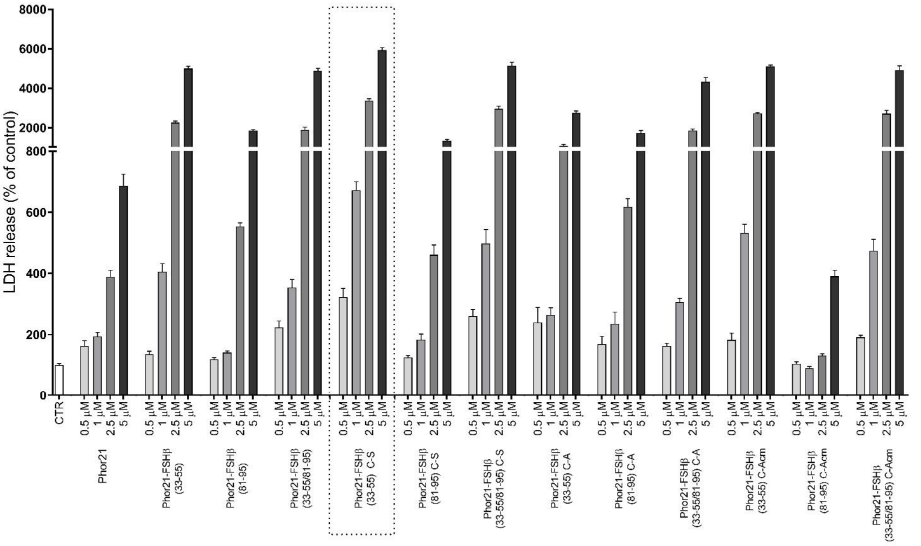

Fig. S3.

Fig. S3. Phor21-FSHβ conjugate variant-mediated cytotoxicity in HEK293-FSHR cells – screening for the most cytotoxic conjugate. The values are the means ±SEMs of three independent experiments (n=8/experiment) in three different passages of the cell line.

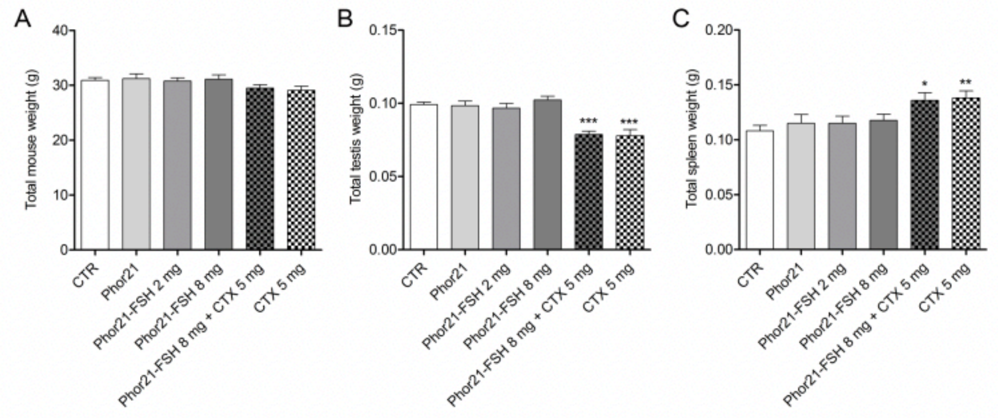

Fig. S4.

Fig. S4. Total body, testis and spleen weights of Phor21-FSHβ33-53C/S (Phor21-FSHβ) conjugates and CTX-treated HEK293-FSHR xenografts. Each bar represents the mean value of the total weight measured at necropsy (n=8–12). Asterisks indicate significant differences (*P≤0.05, **P≤0.01, ***P≤0.01) between the control and treated groups.

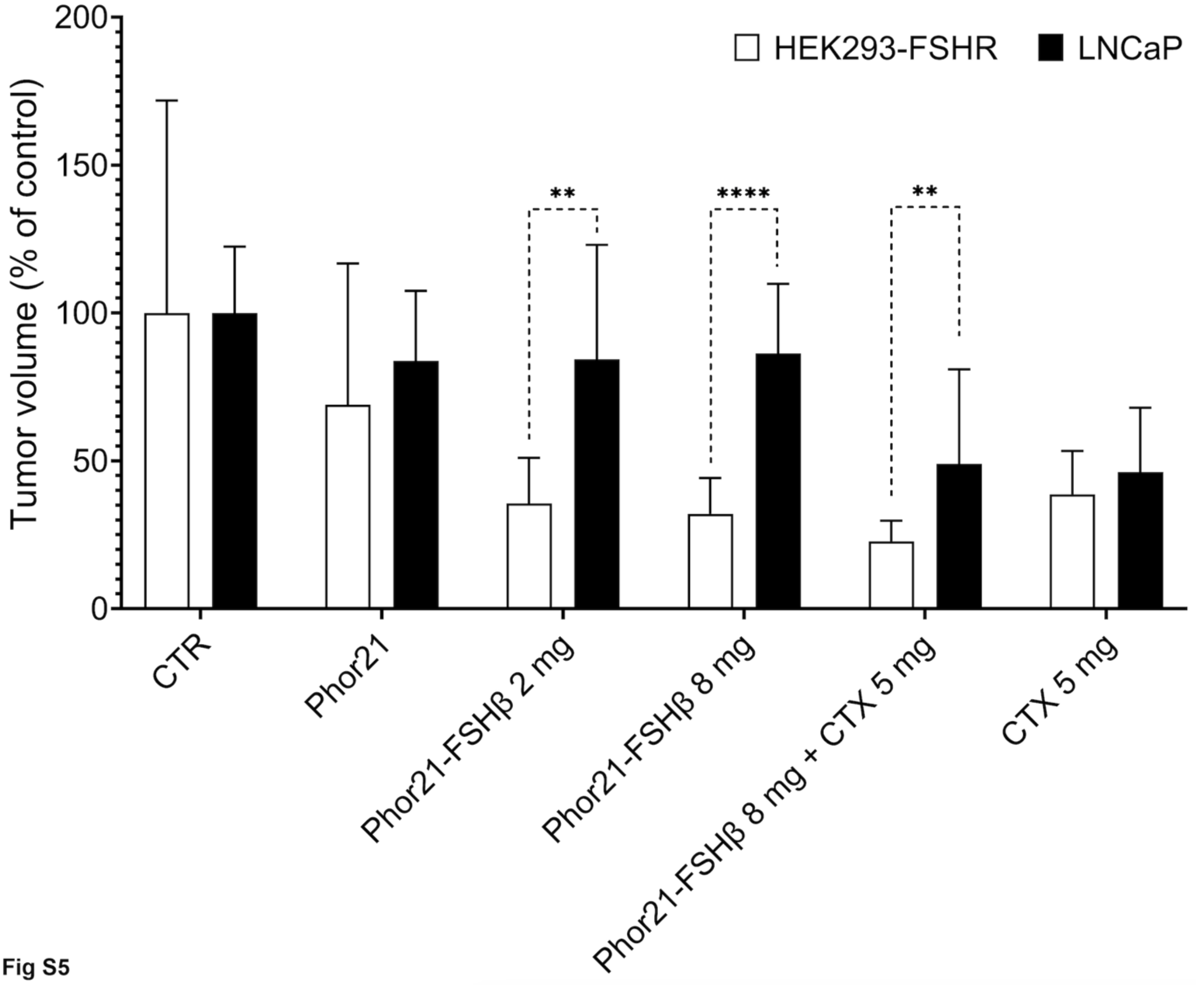

Fig S5

Fig. S5. Effects of Phor21-FSHb33-53C/S (Phor21-FSHβ) and cetrorelix (CTX) treatments on tumor growth (xenograft) (% of control) in HEK293-FSHR and LNCaP cells. Asterisks indicate significant differences between the CTR and the treatments or indicated groups (*P≤0.05; **P≤0.01; ****P≤0.001).

Cetrorelix, CTX; CTR, control treated with vehicle; LNCaP, human prostate cancer cell line; HEK293-FSHR, HEK293 cells stably transfected with the FLAG-hFSHR/pcDNA3.1 expression plasmid

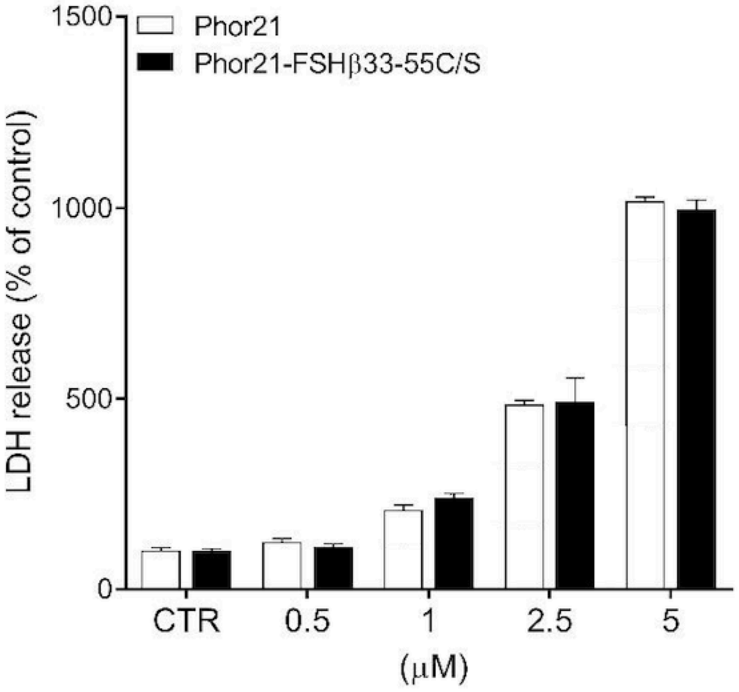

Fig S6

Fig. S6. Comparison of Phor21 and Phor21-SHβ33-53C/S (Phor21-FSHβ) cytotoxicity in HEK-293 cells. The values are presented as the means ±SEMs of three independent experiments (n=8/experiment) in three different passages of the cell line. Asterisks indicate significant differences (**P≤0.05; ***P≤0.001) between the indicated groups.

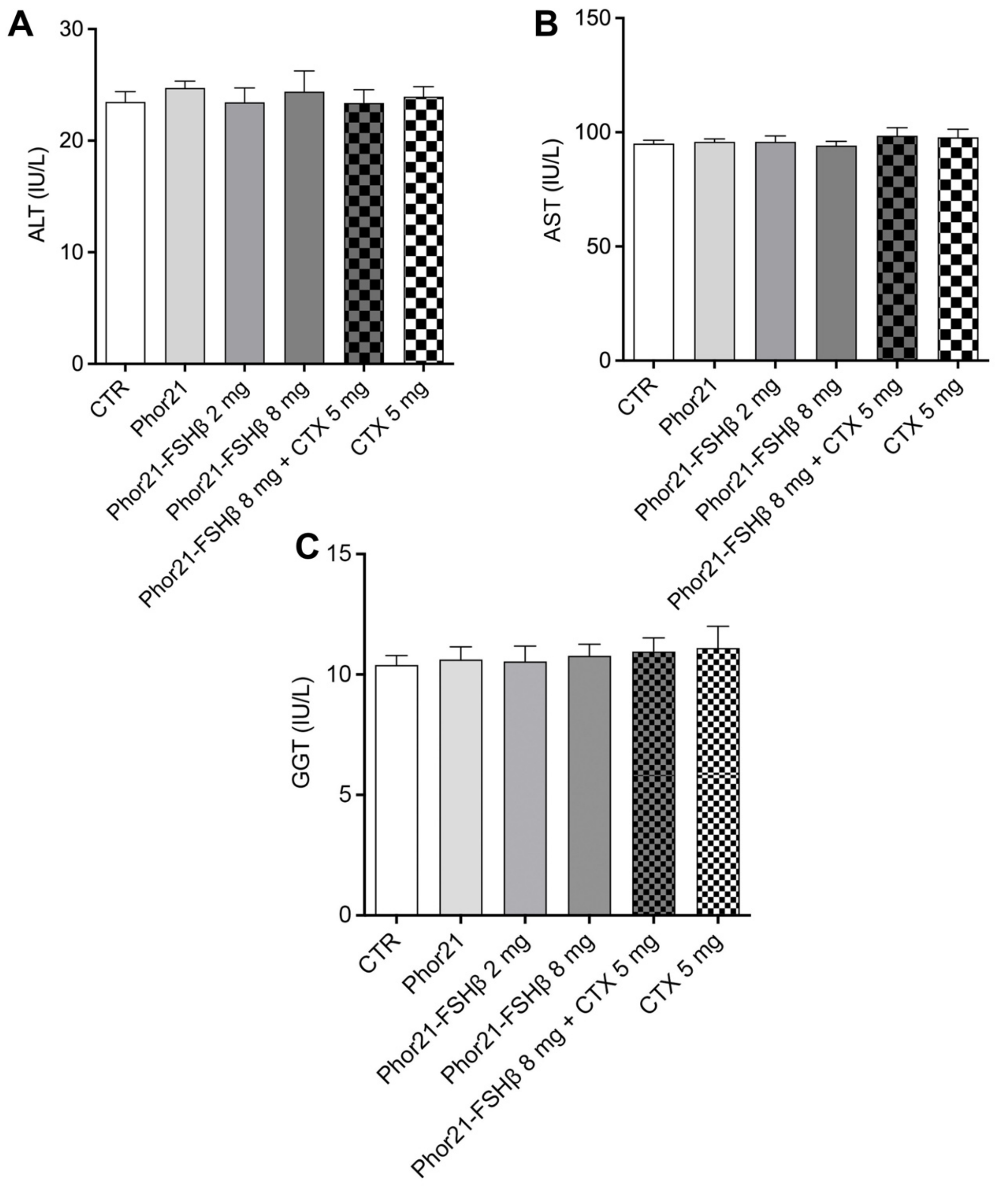

Fig S7 Effects of Phor21-FSHb33-53C/S (Phor21-FSHβ) and cetrorelix (CTX) treatments in HEK293-FSHR xenografts mice (n=8-9) on selected serum biochemical enzymatic parameters.

Biochemical serum enzymatic paremeters of alanine aminotransferase (ALT, U/L)) (A), aspartate aminotransferase (AST, U/L) (B) and glucose, γ-glutamyltransferase (GGT, U/L) (C).

Uncropped full gel picture of Fig S1

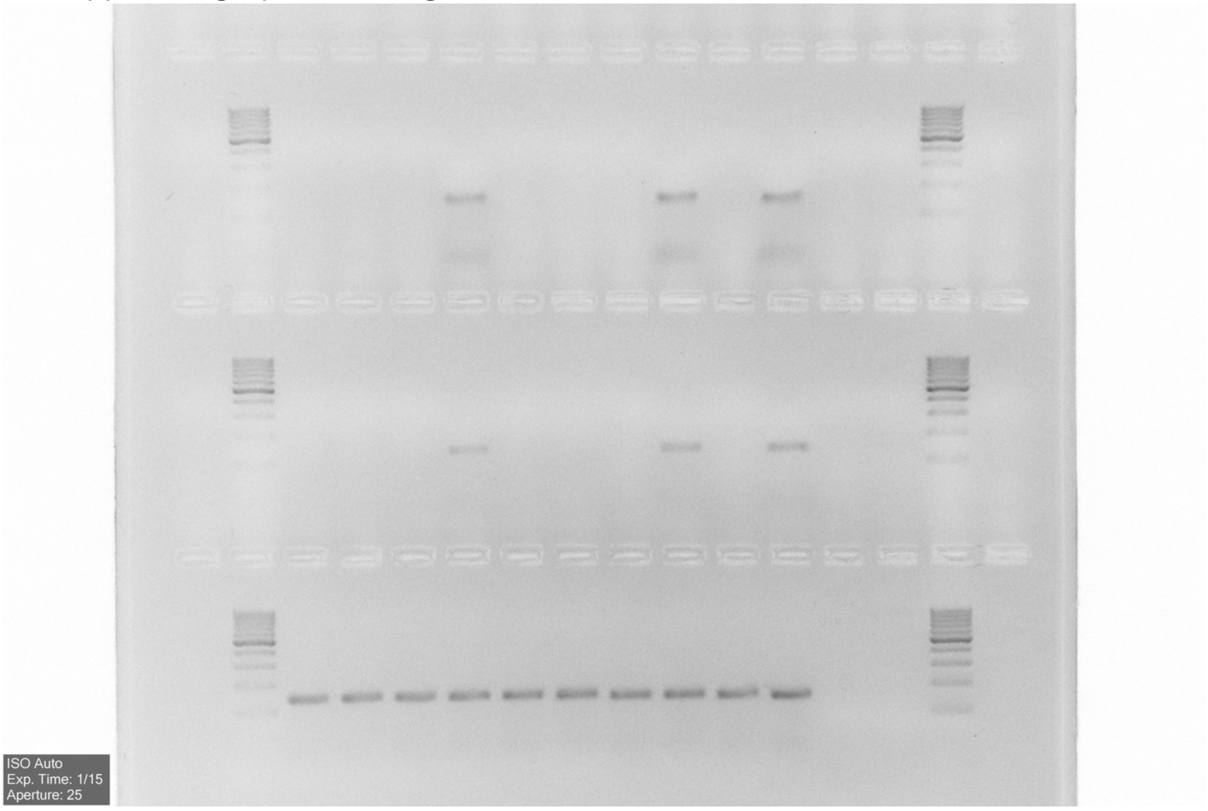

Uncropped full picture of Fig 3C

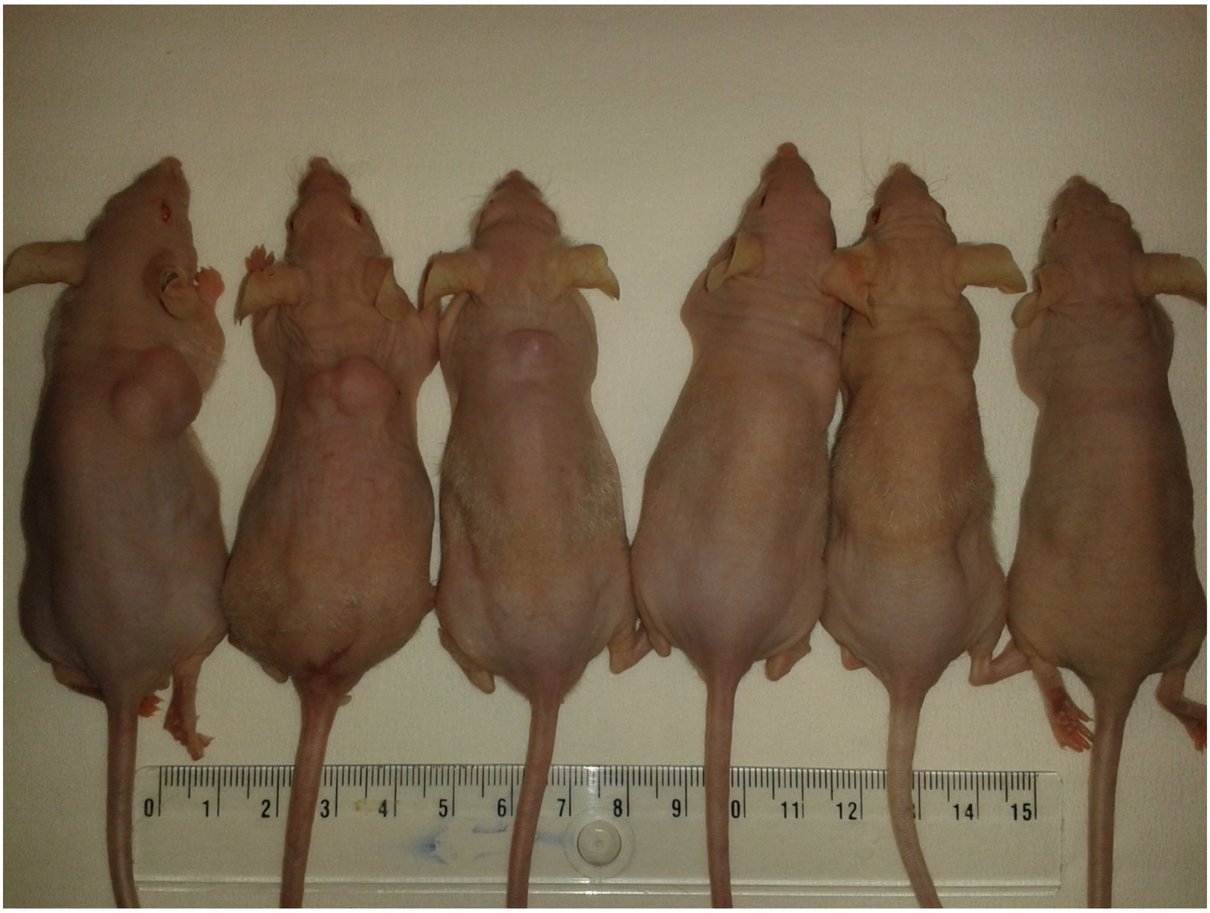

Uncropped full gel picture of Fig 5A

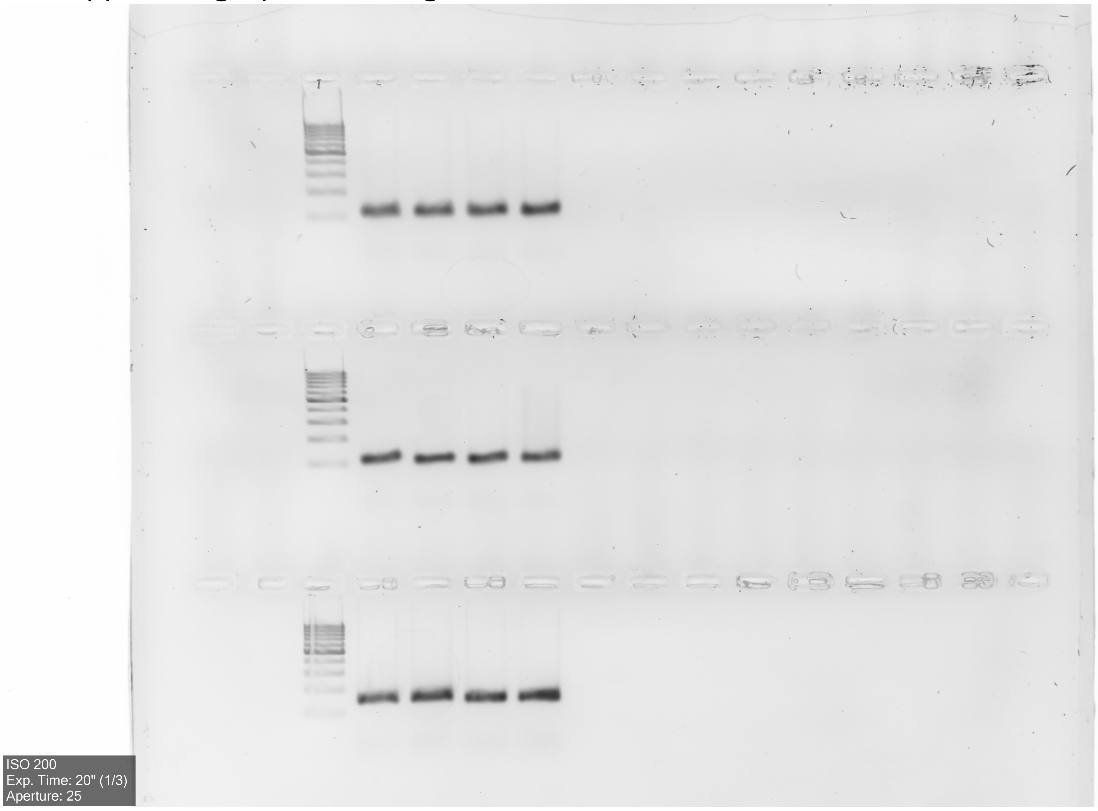

Supplement: Supplementary file 1 — Supplementary Material 1. [file 10020_2025_1292_MOESM1_ESM.pdf]
